# Behavioural adaptation of 158 rescued dogs from abroad during the first six months after adoption in Germany

**DOI:** 10.1038/s41598-026-62598-w

**Published:** 2026-07-30

**Authors:** Lisa Hoth-Zimak, Janina Kickstein, André Klima, Henriette Mackensen, Esther Müller, Helen Louton, Dorothea Döring

**Affiliations:** 1 Chair of Animal Welfare, Ethology, Animal Hygiene and Husbandry, Veterinary Faculty, Department of Veterinary Sciences, Ludwig-Maximilians-Universität München, Munich, Germany; 2 German Animal Welfare Federation, Academy for Animal Welfare, Neubiberg, Germany; 3 Statistical Consulting Unit StaBLab, Department of Statistics, Ludwig-Maximilians-Universität München, Munich, Germany

**Keywords:** Dog, Behaviour, Adoption, Import, Shelter, Animal welfare, Psychology, Psychology, Zoology

## Abstract

In several European countries, adopting rescued dogs from abroad is common, but the dogs’ behaviour has rarely been studied. This first prospective study involved five telephone interviews with 151 owners of 158 dogs from 18 countries. The first interview, collecting each dog’s preliminary data given to the owner by the animal welfare organisation, took place before or few days after adoption to households in Germany, the subsequent ones at one, six and twelve weeks and six months post-adoption. Specific behaviour categories were classified into behaviour scales, and a personality score consisting of 18 behaviour scales was created for each dog and time point. The personality score improved significantly over six months. Age, sex, size, region of origin and type of placement (direct, via shelter/foster home) of the dog and dog experience and residential area of the owner did not significantly influence the personality score. Significantly higher personality scores were determined for dogs housed with conspecific and those without external dog training. Six months after adoption, 79.9% of 149 owners said they would adopt another dog from abroad in future. Our study shows that most dogs adapted well to their new lives, but owners should anticipate behavioural problems, particularly fear-related behaviour.

**Supplementary Information:**

The online version contains supplementary material available at 10.1038/s41598-026-62598-w.

## Introduction

According to estimates, there are 700 million dogs worldwide^1^. Free-roaming dogs, whether with or without owners, make up the largest part (75.0%, about 525 million animals)^1^. The management of these animals (‘dog population management’) varies. In some countries, the killing or lifelong sheltering of free-roaming dogs is required by law^2^, whereas experts often recommend neutering and rereleasing them^3,4^. Indeed, free-roaming dogs are perceived as a danger because they can cause traffic accidents and bite injuries and transmit diseases such as rabies^5,6^. In many regions, private persons or animal welfare organisations take care of these animals. Some of them transport the dogs for adoption reasons to other countries. In 2022, about half (94,000) of all dogs that were transported across Europe and registered via the Trade Control and Expert System (TRACES) were destined for Germany^7^. Most of these came from Romania (42,383), Spain (12,252) and Hungary (11,462)^7^. The adoption of rescued dogs from abroad is popular but also viewed critically. Various experts consider a sustainable, holistic and long-term approach to be the most effective solution to the problem of stray dogs’ management, recommending reproduction control of the dogs (through neutering), public education and much more^3,4^. The export of dogs is not part of this solution, and critics in importing countries have two major concerns: accompanying infectious diseases and behavioural problems of the dogs. The issue of imported diseases has been researched extensively in various studies^8–11^. Although the occurrence of behavioural problems has been a recurring topic of discussion for several years^12,13^, there is little scientific data on the behaviour of rescued dogs, especially in German-speaking countries.

Few studies to date have examined the behaviour of former stray dogs and imported rescued dogs in private households. All of them were retrospective online surveys^14–17^. Dogs from Southern and Eastern Europe showed comparatively positive behavioural traits and a ‘favourable’ character^14^. Demirbas et al^15^. found that most adopted formerly free-roaming Turkish dogs showed behavioural changes within six months, usually viewed positively, and these changes were unrelated to age, sex or origin of the dogs. The owners in the study by Munkeboe et al^16^. similarly reported good adaptation of imported street dogs in Denmark. These studies also recognised behavioural problems or unwanted behaviour in rescued dogs from abroad, such as fear of noises^15–17^ or objects^15–17^, aggressive behaviour^14,15^ and separation problems^15–17^.

To our knowledge, the present study is the first to use a prospective, longitudinal approach to investigate the behaviour of rescued dogs from abroad via telephone interviews. This publication includes the data from five telephone interviews starting pre-adoption and ending six months after adoption. Our study was designed and modified based on our previous studies on laboratory dogs^18,19^. Döring et al^18,19^. examined the behaviour of 145 laboratory dogs before and 12 weeks after their adoption into private households via two behaviour tests and two telephone interviews. A personality score was created for each dog, and the telephone interviews revealed a significant, positive development towards desirable and relaxed behaviour^18,19^. The present study highlights the behavioural adaptation of rescued dogs from abroad in their first six months after adoption in Germany, compares these results with those from the laboratory dogs and contributes to the ongoing research about behavioural problems and integration of adopted dogs from abroad.

Partly based on existing scientific findings, we formulated the following hypotheses for a comprehensive statistical analysis:

1. **Improvement of mean values of personality scores and behaviour scales**: There is a significant improvement in these scores and scales between the telephone interviews conducted one week (1w.) and six months (6mos.) after adoption^15,18,19^.
2. **Direct placement vs. indirect placement (via German shelter or foster homes)**: Dogs that were placed via a German animal shelter or a German foster home have a significantly higher (i.e., more favourable) mean personality score than dogs that were placed directly at all telephone interview times (1w., 6w., 12w., 6mos.). They also show a greater improvement in the mean personality score between the telephone interviews conducted one week (1w.) and six months (6mos.) after adoption.
3. **Placement process**: Dogs with a low score in the ‘placement process’ have a lower mean personality score at all telephone interview times (1w., 6w., 12w., 6mos) than dogs with a high score in the ‘placement process’. They also show a greater improvement in the mean personality score between the telephone interviews conducted one week (1w.) and six months (6mos.) after adoption.
4. **Age**,** sex**,** neutering status**,** region of origin and size of dog and dog experience of owner**: Age^20^, sex^20^, neutering status^20^, region of origin^14^ of the dog and dog experience of the owner^20^ have an influence on the mean personality score at all telephone interview times (1w., 6w., 12w., 6mos) and on the improvement in the mean personality score between the telephone interviews conducted one week (1w.) and six months (6mos.) after adoption. The size of the dog has an influence on the mean personality score at all telephone interview times (1w., 6w., 12w., 6mos.)^21,22^.
5. **Residential area**,** children and other dogs in the household**: Whether the dog lived in a rural or urban^23,24^ area in Germany, whether there were children or grandchildren up to 15 years of age, or whether there were other dogs in the household^25,26^ has an influence on the mean personality score at all telephone interview times (1w., 6w., 12w., 6mos.) and on the improvement in the mean personality score between the interviews conducted one week (1w.) and six months (6mos.) after adoption.
6. **External dog training**: External dog training classes over at least two consecutive telephone interviews have a positive influence on the mean personality score at all telephone interview times (1w., 6w., 12w., 6mos) and on the improvement in the mean personality score between the interviews conducted one week (1w.) and six months (6mos.) after adoption^20^.

## Methods

### Animals

Over a period of 22 months (25 June 2020 to 19 March 2022), data was collected prospectively and longitudinally from 158 rescued dogs from abroad with the help of 151 dog owners in Germany. Five dog owners adopted two dogs and one dog owner three dogs during this period. At the time of participation and the initial telephone interview, the dogs had either not yet been adopted or had been with their new owners for a maximum of seven days. These rescued dogs were former street dogs or dogs from killing stations, animal shelters or rescue centres in their country of origin. Dogs that had been with their new owners for more than a week or that had been imported by private persons or the owners themselves, e.g. from holiday, were not included in the study. Dogs with all sorts of placements participated in the study: they were transferred directly from the animal welfare organisation to the new owner (*n* = 127) or were placed indirectly via a German foster home (*n* = 22) or German animal shelter (*n* = 9). The dogs were placed via 125 German and foreign animal welfare organisations. Twenty-two animal welfare organisations were represented by three to five dogs each. More than half (60.8%) of the 158 dogs were female (*n* = 96, of which 60.4% were neutered) and 39.2% were male (*n* = 62, of which 74.2% were neutered). Most of the dogs adopted were estimated young (up to two years, 64.6%, *n* = 158), but there were also dogs aged between two and five years (21.9%, *n* = 34) and dogs over five years old (13.6%, *n* = 21).

### Ethics declaration

Our study did not include animal experiments. The method consisted of recurring telephone interviews in which participants were asked about the behaviour of their dogs. At the outset, participants were informed about the study purpose, data protection requirements and their right to withdraw at any time. The procedures for data collection and analysis adhered to all regulatory and ethical standards. Informed consent was obtained from all participants for study participation. The Ethics Committee of the Faculty of Veterinary Medicine at LMU Munich approved this study on 13 November 2019 (file number 184-23-08–2019).

### Recruitment of study participants

The study was advertised on the chair’s own website, social media, in journals (e.g. ‘Deutsches Tierärzteblatt’, ‘HundeWelt’) and via email distribution lists (including official veterinarians, veterinarians from the Society for Animal Behavioural Medicine and Therapy: GTVMT, German animal shelters, students) and notices (e.g. in specialist pet food shops, animal clinics and veterinary practices). Owners were asked to inform the animal welfare organisation about the study so that other adopters from the same association could be included in the investigation if necessary.

### Telephone interviews

The study was a prospective, longitudinal study. Over a period of six months, eight telephone interviews were conducted at predetermined survey times with a time tolerance of seven days. This publication focuses on five interviews, whose timing was based on previous studies (Table 1).

**Table 1:** Overview of the five telephone interviews and reasons for selecting the time points with reference to previous studies.

Study section	Point in time, duration, content	Point in time based on
Telephone interview (*n* = 158 dogs)	Shortly **before** or within the first seven days (before) after adoption of the dog, duration approx. 5–10 min, assessment of the initial situation, including information about the dog’s origin, based on information provided to the dog owner by the animal welfare organisation	Own decision to capture all necessary preliminary information
	**Behaviour of the dog after adoption into new home**	
Telephone interview *(n* = 155 dogs)	**One week (1w.)** after adoption of the dog, duration approx. 20–30 min, assessment of the current situation and background information on the family and living situation and the dog’s behaviour, based on information from the dog owner	Döring et al^18,19^.; Gates et al^27^.
Telephone interview (*n* = 151 dogs)	**Six weeks (6w.)** after adoption of the dog, duration approx. 20–30 min, assessment of the current situation and the dog’s behaviour, based on information from the dog owner	Diesel et al^28^.
Telephone interview (*n* =151 dogs)	**Twelve weeks (12w.)** after adoption of the dog, duration approx. 20–30 min, assessment of the current situation and the dog’s behaviour, based on information from the dog owner	Döring et al^18,19^.; Blackwell et al^29^.; Poulsen et al^30^.
Telephone interview (*n* = 149 dogs)	**Six months (6mos.)** after adoption, duration approx. 20–30 min, assessment of the current situation and the dog’s behaviour, based on information from the dog owner	Gates et al^27^.; Diesel et al^28^.

A first interview took place shortly before or within the first seven days after placement and addressed the initial situation regarding the dog’s origin and previous experiences. A maximum time limit of seven days was set for this interview. This meant that the first interview was ideally conducted before the dog was taken in, but no later than the seventh day after placement; the second interview no later than 14 days after adoption, and so on. If, for example, a dog had already been in its new home for three days, the first interview was conducted immediately and the second interview, where possible, on the seventh day after adoption. On rare occasions, interviews one (before) and two (1w.) took place on the same day. If both interviews had to be conducted on the same day, clear distinctions were made between information from the animal welfare organisation and the owner’s own observations.

All telephone interviews after adoption were modified based on previous studies with laboratory dogs^18,19^. We added questions concerning the size of the dog and a general subjective assessment of the character by the owners. The information about the owners and their homes (Table 2) and questions about various behaviours of the dogs and their reactions to people and animals in the same household, everyday situations and interactions with the owner (Table S1) were taken from the questionnaires from the mentioned studies with laboratory dogs. The data from the first telephone interview (before) was based on information from the animal welfare organisations given to the new owner. In the following interviews (1w., 6w., 12w., 6mos.), the owners described the dogs’ behaviours as they had observed in the meantime between the telephone interviews.

**Table 2:** Signalment of the dogs and background information from the telephone interviews before and one week (1w.) after adoption, in case of variable ‘size’ also from telephone interview 6mos., in case of variable ‘dog training classes’ also from all other telephone interviews (6w., 12w., 6mos.). All these variables were used in the statistical models.

Variable	Description or question asked							No.	%
**Type of placement**	Was your dog in a German animal shelter? Was your dog in a German foster home?							*n* = 158	
Direct	Dog was placed directly from abroad.							127	80.4%
Shelter	Dog was placed via German animal shelter.							9	5.7%
Foster home	Dog was placed via German foster home.							22	13.9%
**Age of dogs**								*n* = 155	
Puppy	< 0.5 years							31	20.0%
Young dog	0.5–2 years							69	44.5%
Adult dog	> 2–5 years							34	21.9%
Old dog	> 5 years							21	13.6%
**Sex of dogs**	Is it a male or a female dog?							*n* = 155	
Male								62	40.0%
Female								93	60.0%
**Neutering status of dogs**	Is the dog neutered?							*n* = 155	
Unknown								1	0.6%
Neutered female dog								54	34.8%
Neutered male dog								47	30.3%
Intact female dog								38	24.5%
Intact male dog								15	9.7%
**Residential area of new owner and dog**	Is the area where you live more rural or more urban?							*n* = 155	
Rural	Owner lives in a rural area.							109	70.3%
City	Owner lives rather urban.							46	29.7%
**Children**								*n* = 155	
Child or grandchild	At least one child or grandchild up to 15 years in the household							28	18.1%
None	No child or grandchild in the household							127	81.9%
**Partner dog**	Are there any other pets?							*n* = 155	
Yes	Another dog present in the household							67	43.2%
No	No other dog present in the household							88	56.8%
**Dog experience of owner**	How many dogs have you had so far?							*n* = 155	
No	Owner never had a dog.							27	17.4%
Yes, previous dog	Owner had at least one dog before.							128	82.6%
**Size of dog**	Definition according to the German Animal Welfare Dog Ordinance					1w. No.	1w. %	6mos. No.	6mos. %
						*n* = 155		*n* = 149	
Small	Height at withers under 50 cm					96	61.9%	74	49.7%
Medium	Height at withers 50–65 cm					55	35.5%	71	47.7%
Large	Height at withers over 65 cm					4	2.6%	4	2.7%
**Dog training classes**		1w. No.	1w. %	6w. No.	6w. %	12w. No.	12w. %	6mos. No.	6mos.%
		*n* = 155		*n* = 151		*n* = 151		*n* = 149	
Yes	Owner takes the dog to dog training classes.	16	10.3%	49	32.5%	56	37.1%	51	34.2%
No	Owner does not take the dog to dog training classes.	139	89.7%	102	67.5%	95	62.9%	139	89.7%

Owing to the COVID-19 pandemic and the associated contact restrictions, the *n*-number varies in some cases. For example, study participants initially had no contact with visitors or were unable to attend dog training classes because these were closed.

### Telephone interview questions

There were a few questions in which owners were asked for their subjective assessment. However, most questions related to very specific behavioural responses and the dog’s body language in order to record the behaviour as objectively as possible (Table S1). For example, we used the following categories for questions about the dog’s behaviour with regard to contact with visitors and passerby:

- Friendly contact: dog walks towards the person in a speedy manner with a relaxed body posture and licks or sniffs or jumps up.
- Cautious contact: dog hesitantly approaches the person with signals of fear, does not jump up, watches person or sniffs or licks.
- Fear and avoidance: dog does not approach the person; dog moves away when the person approaches him/her and shows signals of fear.
- Does something else: dog does not seek contact because he/she is busy doing something else, e.g. stays in his/her place (no change of current behaviour).
- Offensive aggression: dog approaches the person and bares teeth or barks or growls or snaps or bites.
- Defensive aggression: dog bares teeth or barks or growls or snaps or bites when being approached by the person.

The following was given as an example of a signal of ‘fear’: crouched posture and tucked tail.

Each survey covered the period since the last telephone interview. If different behaviours were mentioned in response to a question, the one with the lower scale was always evaluated, regardless of how often it occurred (Table S1). For example, if the dog often behaved in a relaxed manner towards children in the household but once growled at them, the aggressive behaviour was used for calculation.

Dogs often perceive some of their owners’ manipulations as threatening, for example leaning or bending over the dog, carrying the dog in their arms or taking food away from the dog. Before these questions (‘manipulations’: ‘leaning over’, ‘carrying’, ‘taking food away’; Table S1) were asked, the dog owners were informed that these interactions are only of interest to us if they have already been experienced in everyday life and that these situations should not be provoked. We did not evaluate the test section on ‘reaction to unknown noise’ in one case of a deaf dog and the questions concerning behaviour of the dog outdoors in two cases of anxious dogs that did not leave the house over all telephone interviews.

### Behaviour scales assessed in the telephone interviews

Behaviour scales for each parameter (Table S1) were defined according to Döring et al^18,19^. for the various behaviour categories. The ‘manipulations’ category was a score consisting of three situations as mentioned above (‘leaning over’, ‘carrying’, ‘taking food away’ by the owner). The scales ranged from zero to three points. Zero meant undesirable behaviour and three meant desirable behaviour (e.g. no fear, no aggression). The mean values of all dogs in a behaviour category were calculated for each time point (1w., 6w., 12w., 6mos.).

### Personality scores of the dogs assessed in the telephone interviews

A personality score was calculated for each dog at the time of each telephone interview, modified according to Döring et al^18,19^. The personality score was an average of the 18 assessed behaviour scales (‘manipulations’ score consisting of three parameters): ‘isolation’, ‘contact with visitor’, ‘passerby’, ‘reaction to unknown object’, ‘second/subsequent reaction to unknown object’, ‘reaction to unknown noise’, ‘second/subsequent reaction to unknown noise’, ‘care’, ‘examination’, ‘placing collar or harness’, ‘leash behaviour’, ‘unknown dog’, ‘luring’, ‘petting’, ‘walk’ and ‘manipulations’ mean value (‘leaning over’, ‘carrying’, ‘taking food away’ by the owner, see above and Table S1).

The personality score ranged from zero to three points. Zero meant undesirable personality and three meant desirable personality. At least half of the parameters had to be available to calculate the personality scores for the individual dog based on the methodology of the studies with laboratory dogs^18,19^. In our study, there were only a few dogs for which six or seven of 18 components were missing (one dog at 6w., one dog at 12w., one dog at 6mos.). No dog therefore had to be excluded at any point in time. If a situation had not yet occurred, such as ‘contact with visitor’, it was not scored. However, if the dog’s behaviour was the decisive and only reason for a situation not taking place, such as two very anxious dogs that never left the home within six months, the situation ‘walk’ was subsequently scored with zero points.

### Researchers

Two researchers (Lisa Hoth-Zimak: L.H.-Z., Janina Kickstein: J.K.) each supervised 79 participating dogs. All telephone interviews with the assigned dog owners were always conducted by the same researcher.

### Pre-test

The telephone interviews were given to laypeople and veterinarians to read through in advance to check whether the questions and possible answers were phrased in an understandable way. Three test subjects who met the inclusion criteria for the study were interviewed via telephone at the specified times in a test run between December 2019 and June 2020.

### Intra- and inter-rater reliability in the telephone interviews

The telephone interviews before and one week (1w.) after adoption were each recorded in 12 surveys using audio recordings and then evaluated and compared by the researchers (J.K. and L.H.-Z). For the comparison, the specific behaviour categories were evaluated. If deviations were identified immediately after the first evaluation, a uniform procedure was agreed upon. Cohen’s kappa was calculated to assess the inter- and intra-rater reliability in the telephone interviews. The calculation of inter-rater reliability yielded a mean value of κ = 0.99 for telephone interview before and κ = 0.98 for telephone interview 1w. According to Landis and Koch^31^ ‘almost perfect agreement’ was achieved in both cases. Intra-rater reliability was also very high, with mean values of κ = 0.99 (J.K.) and κ = 0.97 (L.H.-Z.) for telephone interview before and κ = 0.96 (J.K.) and κ = 0.97 (L.H.-Z.) for telephone interview 1w.

### Statistical analysis

The information provided by dog owners during the telephone interviews was written down in a printed version of the questions by the researchers and then entered into SoSci 2020 software (SoSci Survey GmbH). The datasets were stored in Excel 2021 (Microsoft) and evaluated descriptively. R 4.1.0 and R-Studio (2025.05.0 + 496) were used for further statistical analyses. The α level was set at 0.05.

Different variables were examined to determine influences on the personality score. These variables were defined according to the signalment of the dogs and background information from Table 2.

The ‘placement process’ category consisted of three components: ‘meeting the dog in advance’, ‘duration of the consultation’ and ‘flow of information’ during the placement process (Table S2). The ‘flow of information’ component consisted of four separate criteria. Each criterion was scored with one point for a ‘Yes’ answer and zero points for ‘No’. To assess the ‘flow of information’, all values were summed and then divided by four to calculate the average. This mean value was then combined with the scores for ‘meeting the dog in advance’ and ‘duration of consultation’. Accordingly, a dog could receive between zero and three points in total. We used a Pearson product–moment correlation to analyse the ‘placement process’.

The ‘residential area’ category was determined based on the owner’s assessment, as some suburbs can be very rural and there are different definitions in Germany when an area is considered a city. For the variable ‘size’, only dogs that were at least 0.5 years old at the time of telephone interview 1w. and whose size was reported identically by their owners in telephone interviews 1w. and 6mos. were included in the calculations. This approach allowed us to exclude dogs that were still growing or that fell into different ‘size’ categories owing to measurement errors by their owners. Owing to this age selection for the variable ‘size’, it was analysed in a separate analysis of variance (ANOVA).

The regions of origin were divided into four categories: Eastern European countries (Russia, Ukraine, Slovakia), Southeastern European countries (Romania, Bulgaria, Serbia, Bosnia, Northern Macedonia, Hungary, Croatia, Cyprus, Greece), Southern European countries (Italy, Spain, Portugal) and ‘other countries’ (Turkey, Morocco, Armenia). For statistical analysis of the variable ‘dog training classes’, we only considered dogs that had undergone dog training classes if this information was provided in at least two consecutive interviews.

We analysed each time point separately rather than using a longitudinal approach. Separate models for each time point allow to estimate time specific relationships for the variables included in our analysis more easily which also fits the explorative character of this study. This approach reduces model complexity by avoiding detailed interaction and autocorrelation structures.

We used one-sample *t*-tests of the difference to evaluate improvement between different time points, two-sided Welch’s *t*-tests (allowing unequal variances) to examine the mean values of the personality scores and the influences of variables. An approximate normal distribution of the mean can be assumed from an *n*-value of > 30. We used linear models to evaluate the relationship of more than one variable at once and analysis of variance (with *F*-tests) to estimate the *p*-values. To control for a false discovery rate, the Benjamini–Hochberg procedure was applied. Both non-adjusted and adjusted *p*-values were reported. Only the adjusted *p*-values were mentioned in the text, figures and discussion, with reference to the detailed point in time.

## Results

### Parameters of the personality score: descriptive analysis

#### Isolation

In the first week, 44.9% of 118 dogs showed separation problems which were defined as ‘dog barks, howls or whines more than three minutes and/or destroys objects in the home’ when left alone. Six months after adoption, this proportion had fallen to around a quarter of the dogs (23.9% of *n* = 146; Table S1).

#### Contact with visitor and passerby

After the first week in their new homes, 45.2% of 126 dogs greeted visitors ‘friendly’ (Table S1), increasing to about 52.8% of 144 dogs after six months. Fearful or cautious approaches decreased over time, whereas ‘offensive aggression’, defined as ‘dog approaching the person and baring teeth or barking or growling or snapping or biting’, tripled over the time of the study from 4.7% (of *n* = 126) to 15.3% (of *n* = 144). For up to 44 of the dogs, the owners noticed a difference between visitors. These dogs were for example more fearful towards men and children (Table S1).

Towards passersby, around one-third of the dogs consistently showed ‘friendly’ behaviour. Often dogs ‘did something else’, and a notable proportion showed ‘fear and avoidance’, with these fearful reactions decreasing over time (1w.: 26.4% of *n* = 144, 6mos.: 13.7% of *n* = 146). Aggressive responses towards passersby were rare. The owners noticed a difference in behaviour towards passersby in about one-third of the dogs. These dogs showed fearful or aggressive behaviour particularly towards men.

#### Reaction to unknown object and noise

When encountering unknown objects, about one-third of the dogs initially reacted either ‘relaxed’ or ‘got startled’ and about a quarter were ‘frightened’ for longer than 30 s (Table S1). After six months, ‘relaxed’ reactions became more common, and fear responses decreased. In their second reaction, around half of the dogs ‘made contact’ with the object and the rest either remained ‘relaxed’ or ‘moved back and/or stayed at a distance, showed signals of fear e.g. trembling, crawling or tucked tail’. These proportions changed little over time.

Reactions to unknown noises followed a similar pattern (Table S1). At first, most dogs were ‘relaxed’ or ‘got startled’ and about a quarter were ‘frightened’. Over six months, startle and fear responses declined, and relaxed behaviour increased. In the second reaction, more dogs appeared ‘relaxed’ and fewer ‘made contact’ with the source of noise or showed ‘fear and avoidance’.

Owners also reported that about half of the dogs were afraid of certain objects, especially vacuum cleaners, brooms and dustbins. Many calmed down mostly after two minutes, some of them only when the source was removed or turned off. Likewise, around half were afraid or uncomfortable when hearing certain noises such as loud bangs, gunshots, traffic sounds or sirens, though most dogs settled again within two minutes (Table S1).

#### Manipulations: leaning over, carrying, taking food away by the owner

Owners reported that around three quarters of the dogs tolerated ‘leaning over’, ‘carrying’ and ‘taking food away’ by them during the first week (Table S1). Most still showed this ‘acceptance’ after six months. Some dogs ‘moved away’, showed ‘slight withdrawal’, tried to ‘free’ themselves or displayed aggressive signals. Over six months, all these behaviours decreased in response to ‘leaning over’. However, ‘slight withdrawal’ and ‘aggression’ increased over six months when dogs were carried or when their owners took food away.

#### Behaviour during care and examination by the owner

Most dogs tolerated being brushed and having their paws and teeth examined by their owner, with ‘acceptance’ increasing slightly over the first six months. Around one-fifth showed ‘slight withdrawal’, and only a small number attempted to ‘move away’, ‘freed’ themselves or showed ‘aggression’.

#### Placing collar or harness and leading the dog on a leash

After one week, around two-thirds of the dogs tolerated having a collar or harness put on by their owner, increasing to 82.4% (of *n* = 148) after six months. ‘Slight withdrawal’ and ‘moving away’ became less common over time, and aggressive reactions were rare.

When walking on a leash, only about one-third of the dogs initially ‘followed along’, increasing to over half after six months. Around one-third pulled on the leash. Some dogs could initially only be persuaded to go for a walk by their owner pulling on the leash (1w.: 14.8% of *n* = 155). After six months, this proportion had fallen to 7.4% (of *n* = 149). For almost one-fifth of the dogs (1w.: 17.4% of *n* = 155), the owners stated that ‘even after pulling on the leash, they could not make the dog walk, or the dog stopped, sat or lay down’.

#### Reaction to unknown dog

Around one-third of the dogs initially showed ‘friendly contact’ towards unknown dogs (Table S1). After six months, this proportion rose to about half of the dogs. ‘Cautious’ behaviour and ‘fear and avoidance’ were seen in roughly a quarter at first, and both reactions became less common over time. ‘Offensive aggression’ towards other dogs decreased slightly, and ‘defensive aggression’ increased a little.

#### Luring and petting the dog by the owner

After one week, about half of the dogs ‘came directly’ to their owners when called, increasing to roughly two-thirds after six months. Some ‘approached slowly and cautiously’, and a small number ‘did not come’ at all, and these behaviours decreased after six months. Most dogs ‘enjoyed’ being petted. A few dogs ‘tolerated the situation with tensed body’ (i.e. ‘acceptance’), ‘tried to withdraw (movement of head or body)’ (i.e. ‘slight withdrawal’), ‘moved away so the owner could not pet the dog’ (i.e. ‘moves away’) or ‘bared their teeth or barked or growled or snapped or bit’ (i.e. ‘aggression’).

#### Behaviour during walk

On walks, just under half of the dogs were ‘mostly calm’ after one week, around one-fifth were ‘excited’, and one-third ‘frequently showed signs of fear’. After six months, both ‘excited’ and ‘anxious’ behaviour had halved, and about three quarters of the dogs appeared ‘relaxed’.

#### Parameters of the personality score: mean values of the behaviour scales

**Fig. 1:**
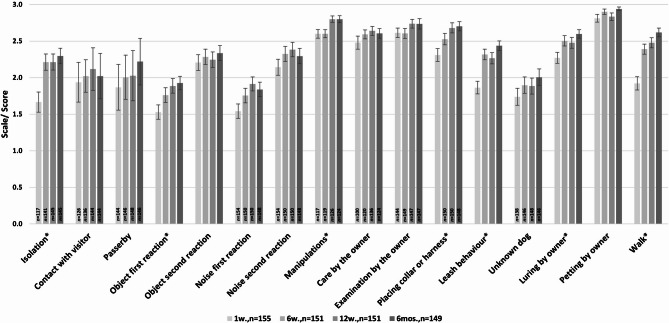
Comparison of the mean values of the behaviour scales (‘manipulations’ score consisting of three behaviour scales) and standard errors at all telephone interview times (1w., 6w., 12w., 6mos.) after adoption. Scales ranged from zero (undesirable) to three (desirable behaviour) points (for scales definition see Table S1). Reference number: 1w.: *n* = 155, 6w.: *n* = 151, 12w.: *n* = 151, 6mos.: *n* = 149, with deviating reference numbers indicated on columns; ‘*’ indicating significant improvement between 1w. and 6mos.

The dogs improved in most of the mean values of the behaviour scales and the ‘manipulations’ score between week one and month six after adoption (Fig. 1). In the categories ‘contact with visitor’, ‘reaction to unknown noise’ and ‘care’, the mean value decreased slightly between week twelve (12w.) and month six (6mos.). The initial reactions to unknown noises and objects and the dogs’ behaviour towards unknown dogs had the lowest scales. In the categories ‘isolation’ and ‘walk’, the mean value increased the most (+ 0.6 and + 0.7 points, respectively) between the first week (1w.) and the sixth month (6mos.). The improvement from week one to month six was statistically significant in the categories ‘isolation’, ‘reaction to unknown object’ (first reaction), ‘manipulations’ score, ‘placing collar or harness’, ‘leash behaviour’, ‘luring’ and ‘walk’ (Table S3).

#### Mean values of the personality scores

**Fig. 2:**
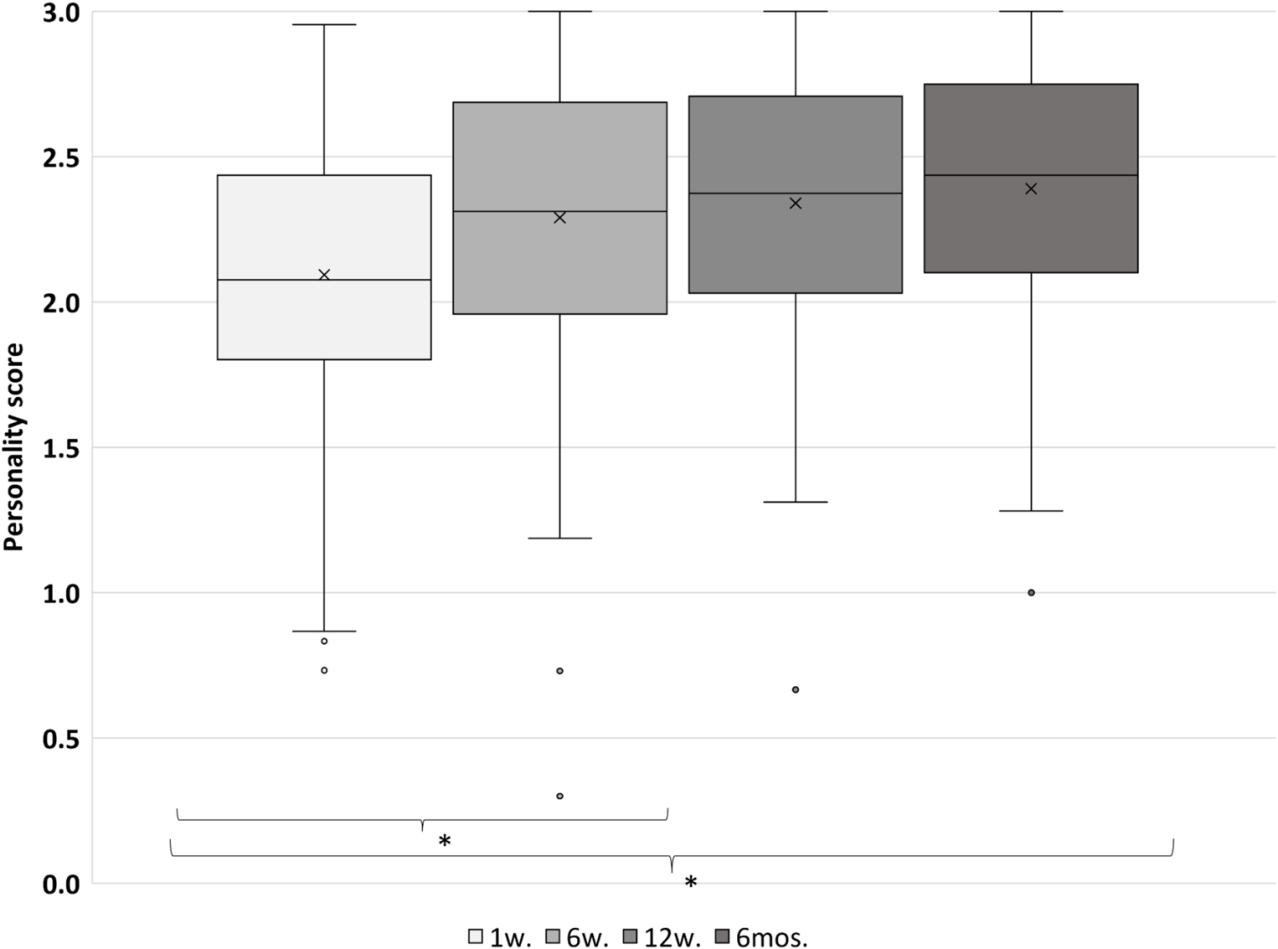
Boxplots of the personality scores at all telephone interview times after adoption (1w., 6w., 12w., 6mos.), with the median indicated by a line and the mean value by ‘x’. Personality scores included a mean value from 18 behaviour scales and ranged from zero (undesirable) to three (desirable behaviour) points. Improvement between 1w. and 6w. and between 1w. and 6mos. was statistically significant, indicated by ‘*’.

The mean values of the personality scores increased from 2.1 to 2.4 between the telephone interviews after the first week and the sixth month (Fig. 2). The improvement between week one (1w.) and week six (6w.: *p* < 0.001) and between week one (1w.) and month six (6mos.: *p* < 0.001) was statistically significant (Table S4). The mean value of the difference was 0.3 points. Our hypothesis was thus confirmed. At six and twelve weeks, the mean value was 2.3. The first six weeks contributed the most to the overall average improvement of 0.3 points (0.2 points, see Table S4 for the performed tests and results).

About two-thirds of the dogs showed the same or a higher personality score as compared with the previous interview. The progression of personality scores for dogs for which all four values were available is shown in Figure S1. Two dogs stand out as outliers with very low personality scores. Both dogs did not leave the house at all during the entire six months because of fear (Fig. S1).

### Variables

#### Placement via German shelter and foster home vs. direct placement

Dogs that were adopted directly from the animal welfare organisation had a higher mean personality score over all interview times than dogs placed via shelters or foster homes (Table 3). The difference was not statistically significant. Our hypothesis could not be proven.

**Table 3:** Comparison of mean personality scores between direct placement and indirect placement via shelter or foster home at all telephone interview times (1w., 6w., 12w., 6mos.) and difference between week one (1w.) and month six (6mos.). A Welch two-sample *t*-test was used for calculation with the alternative hypothesis: ‘true difference in means between group direct placement and group German shelter or foster home is not equal to zero’ (df, degrees of freedom).

Telephone interview	t-value	df	*p*-value	*p*-value adjusted	Mean personality score	
					Direct placement	Placement via shelter or foster home
1w.	1.610	55.18	0.113	0.261	2.120	1.988
6w.	1.742	52.49	0.087	0.228	2.322	2.169
12w.	2.068	50.29	0.044*	0.161	2.376	2.204
6mos.	1.857	46.67	0.070	0.210	2.423	2.261
Difference in points of mean personality score between 1w. and 6mos.	0.288	45.91	0.774	0.844	0.305	0.280

*Significant with *p* < 0.05.

#### Placement process

Dogs with low scores in the ‘placement process’ category did not have significantly lower personality scores at any telephone interview time (Table 4). The difference between week one (1w.) and month six (6mos.) was also not statistically significant. Our hypothesis could not be proven. On the contrary, we observed a small negative correlation: dogs with a high score in the ‘placement process’ category tended to have a slightly lower personality score, albeit without statistical significance.

**Table 4:** Analysis of the variable ‘placement process’ with regard to the mean personality score at all telephone interview times (1w., 6w., 12w., 6mos.) and difference between week one (1w.) and month six (6mos.). A Pearson product–moment correlation was used for calculation with the alternative hypothesis: ‘true correlation is not equal to zero’ (df, degrees of freedom; Cor, correlation).

Telephone interview	t-value	df	*p*-value	*p*-value adjusted	Cor
1w.	−1.076	153	0.284	0.473	−0.087
6w.	−1.190	149	0.236	0.422	−0.097
12w.	−1.015	149	0.312	0.485	−0.083
6mos.	−0.697	147	0.487	0.671	−0.057
Difference 1w. to 6mos.	0.544	147	0.587	0.750	0.045

#### Age, sex, neutering status, region of origin of dogs and dog experience of owners

Old dogs started with the highest mean personality score (2.29) in comparison with all age groups in week one and had the smallest difference between week one and month six (+ 0.21 points; Table 5). Neutered male dogs had higher mean personality scores than the other groups over all interviews, followed by intact female dogs, neutered female dogs and intact male dogs. Intact female dogs showed the biggest improvement between week one and month six (+ 0.41 points; Table 5).

Dogs with owners who had dog experience, defined as previous ownership of a dog, showed higher personality scores over all telephone interview times than dogs with unexperienced owners (Table 5). Dogs from ‘Eastern Europe’ and dogs from ‘Southeastern Europe’ showed lower mean personality scores whereas dogs from ‘Southern Europe’ and ‘other countries’ showed the highest mean personality scores (Table 5). Dogs from ‘other countries’ showed the biggest (+ 0.41) and dogs from ‘Southern Europe’ the smallest (+ 0.13) improvement between week one and month six (Table 5).

These observed differences concerning the age, sex, neutering status and region of origin of the dog or the dog experience of the owners were not statistically significant (Table 6). Our hypothesis regarding age, sex, neutering status, region of origin and dog experience could not be proven (Table 6).

#### Size of the dog

Medium-sized dogs showed the highest mean personality scores over all telephone interview times (Table 5). Large dogs had the lowest mean personality score after six months and the smallest improvement between week one and month six. These differences were not statistically significant (Table 6). Our hypothesis could not be proven.

**Table 5:** Variables used in statistical analysis (see Table 6), mean values of the personality score at all telephone interview times (1w., 6w., 12w., 6mos.) after adoption and difference in mean values between week one and month six.

Variable	Description	Mean personality score				
		1w.	6w.	12w.	6mos.	Difference 1w. to 6mos.
Age of the dog		*n* = 155	*n* = 151	*n* = 151	*n* = 149	
Puppy	< 0.5 years	2.03	2.31	2.31	2.32	0.29
Young dog	0.5–2 years	2.08	2.34	2.36	2.43	0.35
Adult dog	> 2–5 years	2.05	2.26	2.36	2.35	0.30
Old dog	> 5years	2.29	2.14	2.28	2.45	0.21
**Sex and neutering status of the dog**		*n* = 154	*n* = 150	*n* = 150	*n* = 148	
F	Female, intact	2.00	2.29	2.36	2.41	0.41
Fn	Female, neutered	2.07	2.26	2.29	2.37	0.30
M	Male, intact	2.10	2.22	2.29	2.32	0.22
Mn	Male, neutered	2.22	2.38	2.41	2.44	0.22
**Dog experience of owner**		*n* =155	*n* = 151	*n* = 151	*n* = 149	
No	No previous ownership of a dog	2.02	2.11	2.27	2.30	0.28
Yes	Previous ownership of a dog	2.11	2.33	2.36	2.41	0.30
**Region of origin** ^**1**^		*n* = 155	*n* = 151	*n* = 151	*n* = 149	
Southeastern Europe		2.03	2.25	2.29	2.37	0.34
Southern Europe		2.30	2.39	2.48	2.43	0.13
Eastern Europe		2.00	2.12	2.26	2.23	0.23
Other countries		2.24	2.61	2.52	2.65	0.41
**Size of the dog** ^**2**^		*n* = 155	*n* = 151	*n* = 151	*n* = 149	
Small	Height at withers under 50 cm	2.02	2.23	2.29	2.32	0.30
Medium	Height at withers 50–65 cm	2.22	2.38	2.44	2.52	0.30
Large	Height at withers over 65 cm	2.09	2.35	2.29	2.25	0.16

^1^ See methods for definitions.

^2^ Definition according to the German Animal Welfare Dog Ordinance.

**Table 6:** Results of the linear models evaluating the relationship of the variables age, sex, neutering status, region of origin of the dog and dog experience of the owner with the mean personality score at certain time points (1w., 6w., 12w., 6mos.) after adoption. Analysis of variance and *F*-tests (ANOVA, F-Tests) were used for calculation. F-Tests were used for calculation of *p*-values (df, degrees of freedom). Size was evaluated in a separate model owing to age selection (only dogs at least 0.5 years old at telephone interview 1w. were included).

Variable	F-value	df	*p*-value	*p*-value adjusted
Age 1w.	2.189	3	0.092	0.234
Age 6w.	0.436	3	0.727	0.832
Age 12w.	0.256	3	0.857	0.869
Age 6mos.	1.209	3	0.309	0.485
Sex 1w.	4.809	1	0.029*	0.122
Sex 6w.	0.948	1	0.332	0.503
Sex 12w.	0.525	1	0.470	0.658
Sex 6mos.	0.085	1	0.771	0.844
Neutering status 1w.	0.441	1	0.508	0.688
Neutering status 6w.	1.259	1	0.264	0.453
Neutering status 12w.	0.053	1	0.818	0.859
Neutering status 6mos.	0.255	1	0.614	0.750
Region of origin 1w.	2.014	3	0.115	0.261
Region of origin 6w.	2.120	3	0.100	0.247
Region of origin 12w.	1.845	3	0.142	0.288
Region of origin 6mos.	1.737	3	0.162	0.302
Dog experience 1w.	1.033	1	0.311	0.485
Dog experience 6w.	6.213	1	0.014*	0.078
Dog experience 12w.	0.938	1	0.335	0.503
Dog experience 6mos.	1.343	1	0.249	0.436
Sex: neutering status 1w.	0.0383	1	0.845	0.869
Sex: neutering status 6w.	0.0603	1	0.806	0.857
Sex: neutering status 12w.	0.4197	1	0.518	0.691
Sex: neutering status 6mos.	0.3632	1	0.548	0.719
**Improvement between 1w. and 6mos.**				
Age	0.628	3	0.598	0.750
Sex	3.209	1	0.075	0.212
Neutering status	1.527	1	0.219	0.400
Region of origin	1.835	3	0.144	0.288
Dog experience	0.032	1	0.859	0.869
Sex: neutering status	0.286	1	0.594	0.750
**Size** (evaluated in a separate model)				
Size 1w.	0.4868	2	0.616	0.750
Size 6w.	0.2388	2	0.788	0.849
Size 12w.	0.8180	2	0.708	0.832
Size 6mos.	2.3106	2	0.104	0.250

*Significant with *p* < 0.05.

#### Living conditions of the dogs in Germany

Dogs living in rural areas in Germany had a higher mean personality score at all telephone interview times than dogs in urban areas (Table 7). The difference was not statistically significant. Our hypothesis that the place of residence had an influence on the personality score could not be proven.

Children up to 15 years old living in the same household did not have a statistically significant influence on the personality scores (Table 7). Our hypothesis was not confirmed.

Dogs with one or more other dogs in the household had a higher mean personality score at all telephone interview times than dogs without other dogs in the household (Table 7). The difference was statistically significant in week six (6w.: *p* = 0.014) and month six (6mos.: *p* = 0.014). The improvement of both groups was about the same (+ 0.3 points between 1w. and 6mos). Our hypothesis was thus partially confirmed.

Dogs that did not receive external dog training for the time of at least two consecutive telephone interviews showed a higher mean personality score at all telephone interview times and a greater improvement between week one and month six than those that attended dog training classes in the defined time frames (Table 7). The difference was statistically significant in week twelve (12w.) and month six (6mos.), both with *p* < 0.01. Our hypothesis that external dog training would improve the personality score could not be proven.

**Table 7:** Comparison of mean personality scores at all telephone interview times (1w., 6w., 12w., 6mos.) regarding the living conditions of the new owners: rural vs. urban area, with vs. without children in the household, with vs. without other dogs in the household and with vs. without external dog training classes (1w.: *n* = 155, 6w.: *n* = 151, 12w.: *n =* 151, 6mos.: *n =* 149) and difference between week one (1w.) and month six (6mos.). A Welch two-sample *t*-test was used for calculation with the alternative hypothesis: ‘true difference in means between groups is not equal to zero’ (df, degrees of freedom). Dog training classes were defined as participation duration of at least two consecutive telephone interviews.

Variable	t-value	df	*p*-value	*p*-value adjusted	Mean personality score	
Rural vs. urban area					Rural area	Urban area
1w.	1.447	77.343	0.152	0.290	2.130	2.008
6w.	1.978	69.739	0.052	0.175	2.344	2.164
12w.	2.306	78.359	0.024*	0.106	2.395	2.212
6mos.	1.453	84.572	0.150	0.290	2.425	2.311
Difference 1w. to 6mos.	−0.342	79.584	0.733	0.832	0.292	0.319
**Children vs. no children in household**					**No children**	**Children or grandchildren up to 15 years**
1w.	0.162	51.50	0.871	0.871	2.096	2.083
6w.	−0.367	45.37	0.715	0.832	2.285	2.317
12w.	0.945	41.48	0.350	0.516	2.355	2.275
6mos.	1.923	39.93	0.062	0.200	2.418	2.255
Difference 1w. to 6mos.	1.799	38.76	0.080	0.217	0.325	0.173
**Other dogs vs. no other dogs in household**					**No dogs**	**Dogs**
1w.	−2.388	128.58	0.018*	0.089	2.016	2.196
6w.	−3.117	144.06	0.002*	0.014*	2.192	2.425
12w.	−1.840	141.08	0.068	0.210	2.285	2.417
6mos.	−3.113	138.59	0.002*	0.014*	2.299	2.519
Difference 1w. to 6mos.	−0.291	135.27	0.772	0.844	0.291	0.312
**Dog training classes vs. no dog training classes**					**No dog training classes**	**Dog training classes**
1w.	1.988	105.03	0.0495*	0.173	2.147	1.992
6w.	2.041	118.34	0.0435*	0.161	2.346	2.188
12w.	4.199	121.51	< 0.001*	< 0.01*	2.442	2.154
6mos.	3.667	96.66	< 0.001*	< 0.01*	2.487	2.211
Difference 1w. to 6mos.	1.545	95.48	0.126	0.278	0.341	0.223

*Significant with *p* < 0.05.

### Additional parameters from the telephone interviews

#### Playing with and hand-feeding by the owner

‘Playing’ was defined as ‘dog follows the toy and/or picks up the toy with his/her mouth’. After the first week, half of all dogs ‘played’ (49.0% of *n* = 155; Table S1). After six months, this proportion increased to 71.8% (of *n* = 149). Most dogs already took food from their owner’s hand indoors (1w.: 94.2% of *n* = 155). Outdoors, slightly fewer dogs took food from their owner’s hand: 82.2% (of *n* = 152). After six months, almost all dogs took food from their owner’s hand indoors and outdoors (6mos.: 99.3% of *n* = 149 and 93.3% of *n* = 146, respectively).

#### Subjective assessment of the dog’s character

During the first week in their new home, 60.4% (of *n* = 154) of the dogs were described as ‘relaxed’, which meant that the dog stayed mostly calm (Table 8), 6.4% were ‘predominantly excited, constantly running around and panting a lot’ (‘excited’) and 33.1% were assessed as ‘anxious’, which meant they were frequently showing signs of fear (e.g. trembling, crouched posture, tucked tail, attempts to escape). Over six months, the proportion of ‘relaxed’ dogs increased, and ‘excited’ and ‘anxious’ behaviour assessments decreased. Most dogs were reported by their owners as ‘relaxed’ after six months (92.7% of *n* = 149; Table S1).

**Table 8:** Character of the dogs as subjective assessment given by their owners at all telephone interview times (1w., 6w., 12w., 6mos.) and scale (zero to three points) for the behaviour categories.

Character of the dog	Definition: How would you describe your dog?	Scale	1w. *n* = 154		6w. *n* = 151		12w. *n* = 151		6mos. *n* = 149	
			No.	%	No.	%	No.	%	No.	%
Relaxed	Dog is mostly calm	**3**	93	60.4%	129	85.4%	134	88.7%	139	92.7%
Excited	Dog is predominantly excited, constantly running around and panting a lot	**2**	10	6.5%	6	4.0%	7	4.6%	3	2.0%
Anxious	Dog frequently shows signs of fear (e.g. trembling, crouched posture, tucked tail, attempts to escape)		51	33.1%	16	10.6%	10	6.6%	7	4.7%
Frequency of anxiety										
	Dog showed these signs of anxiety up to five times a day	**1**	29	18.7%	12	7.9%	5	3.3%	7	4.7%
	Dog showed these signs of anxiety more than five times a day	**0**	22	14.2%	4	2.6%	5	3.3%	0	0.0%

#### Comparison of subjective assessment of the dog’s character and objective personality score

**Fig. 3:**
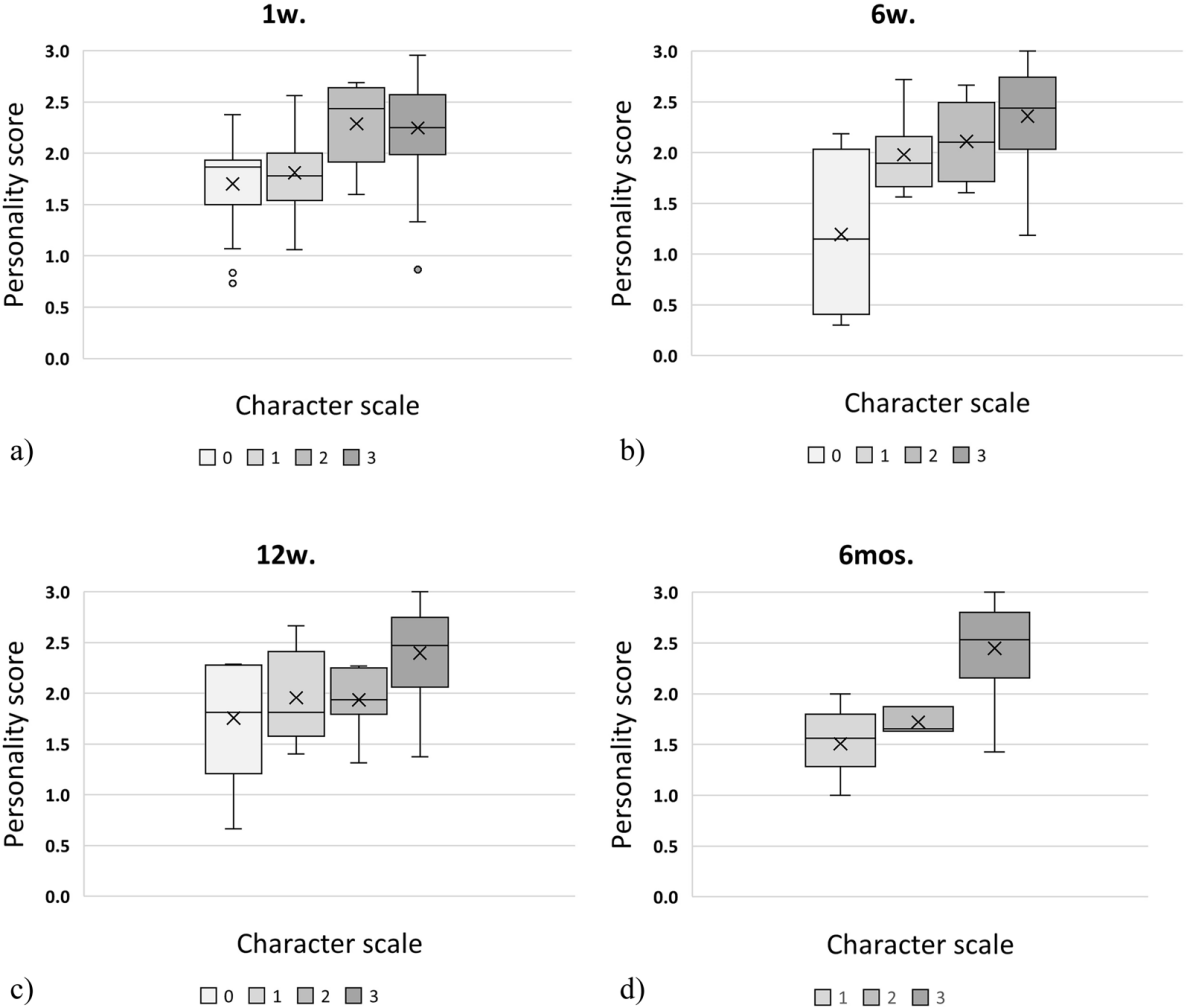
(a-d). Boxplots comparing the personality scores (0–3 points) with the character scales based on the owners’ assessment (0–3 points) at all telephone interview times after adoption (a: 1w., b: 6w., c: 12w., d: 6mos.). There is no character scale ‘0’ six months after adoption. Median indicated by a line and mean value by ‘x’.

To determine whether the subjective assessment aligned with the dogs’ evaluation based on personality scores, we created boxplots (Fig. 3a-d). Despite the differing methodologies, most dogs with a low character rating by their owner also had low personality scores, which increased in a similar manner with higher character points.

#### Would you choose a rescued dog from abroad again for adoption?

Six months after adoption, 119 persons (79.9% of *n* = 149) said in the telephone interview they would agree to adopt another rescued dog from abroad in future (Table S5). Ten dog owners (6.7%) would only adopt a dog from abroad again under certain conditions, for example if they had more information about the dog’s behaviour or health, or if they could meet the dog beforehand (Table S6). Twenty persons (13.4%) said they would not adopt another dog again. The dog owners stated reasons such as the dogs being ‘surprise packages’, lacking socialisation, their health status being unknown, or illnesses being concealed (Table S6).

**Fig. 4:**
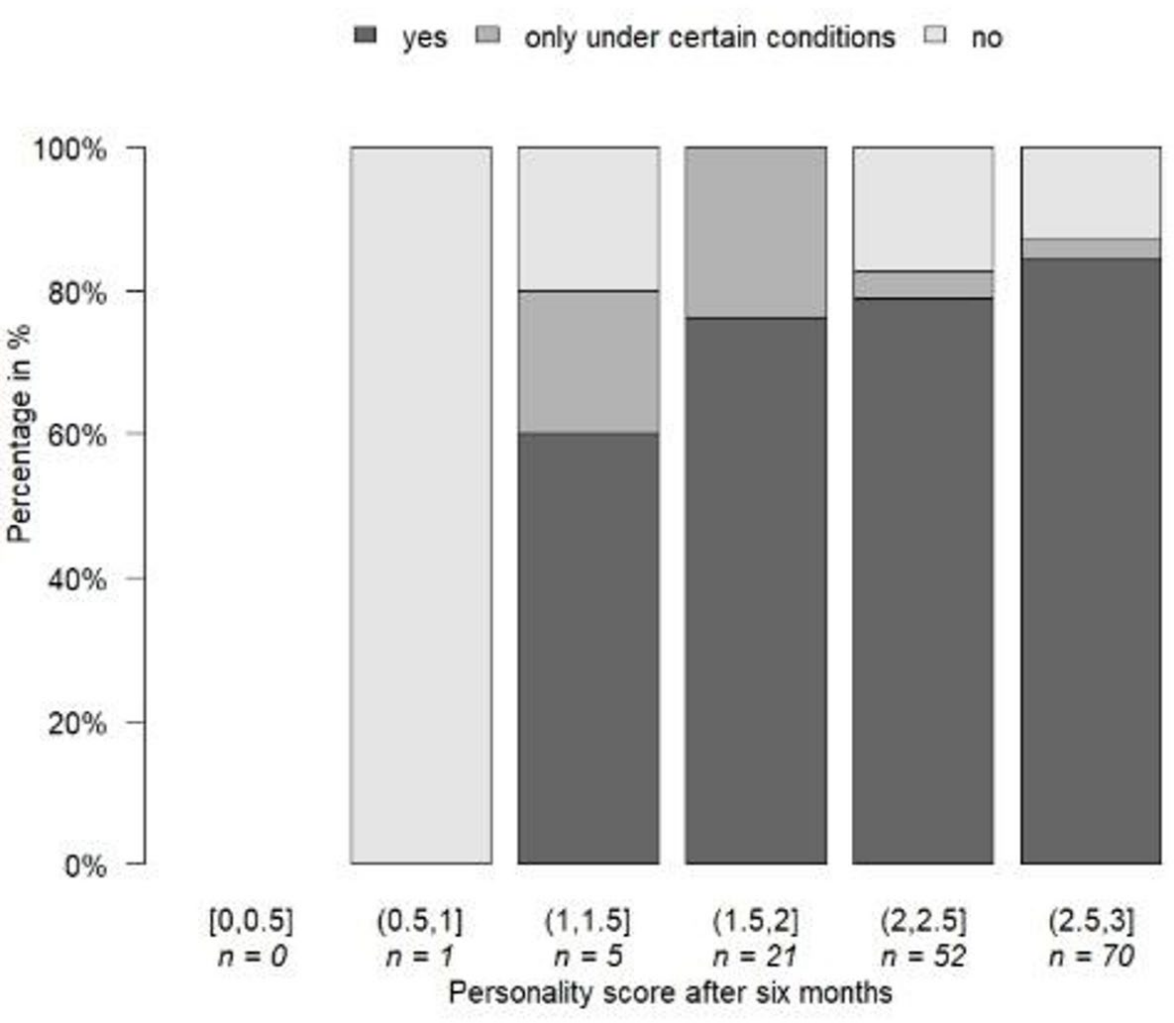
Comparison of the personality scores of the dogs at the time of the last telephone interview (6mos.) with the willingness of the owners to adopt another dog from abroad in future, only under certain conditions or not at all. Personality score ranged from zero to three points.

When comparing the answers given to the question of whether dog owners would choose to adopt a rescued dog from abroad again with the personality score of the current dog at the time of telephone interview 6mos., it was noticeable that even if the dogs had high personality scores (2–3), some owners would decide against adopting a dog from abroad in future (Fig. 4). The higher the personality score of their dog, the more likely owners were to consider adopting another dog from abroad.

## Discussion

The behaviour of the rescued dogs in this study changed over the examined six months after adoption. Especially in the early phase of adoption, the dogs frequently showed fear in various situations, but their personality scores improved significantly from week one to month six. A positive change in the behaviour of adopted stray dogs was also observed in previous studies conducted in Turkey and Denmark^15,16^. In the study by Demirbas et al^15^. 69.3% of 75 owners of former Turkish stray dogs mentioned their behaviour change as positive. The owners in the study by Munkeboe et al^16^. similarly reported good adaptation of imported street dogs in Denmark. Demirbas et al^15^. found the biggest improvement of the dogs’ behaviour assessed retrospectively by the owners in the first six months after adoption. In our prospective, longitudinal study, the personality scores of the dogs showed the biggest improvement within the first six weeks after adoption. Furthermore, our results agree with those of two studies with adopted laboratory dogs^18,19^, on whose (modified) methodology our study was based. In the individual behaviour categories, the laboratory dogs^18,19^ showed a significant improvement in ‘luring’, first and second reaction to an ‘unknown noise’, and ‘leash behaviour’, and the rescued dogs in our study showed a significant improvement in ‘luring’ and ‘leash behaviour’. In addition, we found a significant improvement in the categories ‘isolation’, ‘reaction to unknown object’, ‘placing collar or harness’, ‘walk’ and ‘manipulations’.

The dogs’ sex, neutering status, age, size, region of origin and type of placement (direct or via shelter/foster home) did not have a significant influence on the personality score in our study. In a study involving 34 adopted shelter dogs, behavioural observations were made while the dogs were still in the shelter, and following adoption, a telephone survey of the owners regarding the dogs’ behaviour was conducted^32^. No influence of sex on behaviour was found^32^. Rather than the sex of the dog, the individual character of the dog was relevant^32^. The sex of the laboratory dogs studied by Döring et al^18^. also had no influence on the behaviour. In contrast, male dogs have been considered ‘braver’ than female dogs^20,33,34^, and Kubinyi et al^20^. found that intact dogs were ‘calmer’ than neutered dogs. In a behaviour test before rehoming, the laboratory dogs aged between 1.5 and 2 years showed lower personality scores than puppies and adult dogs (defined as 2.5 years and older)^18^. This finding was not confirmed for our rescued dogs from abroad based on the data collected after adoption.

Whereas the size of the dog had no significant influence on the behaviour of the dogs in our study, Mc Greevy et al^21^. found that undesirable behaviours – with the exception of hunting motivation – increase and are more pronounced the smaller the dog is. According to Martínez et al^22^., smaller dogs display more aggressive behaviour and larger dogs exhibit fewer undesirable behaviours. However, in these studies, the size of the dogs was defined by weight (‘large’: 25–40 kg and > 30 kg). We cannot rule out that some of our ‘medium-sized’ dogs would also fall into this weight category. A comparison is therefore difficult.

In contrast to our non-significant findings regarding the region of origin, Graf et al^14^. observed differences in the behaviour of dogs from Eastern Europe and Southern Europe. Although their country categorisations did not entirely match ours, Graf et al^14^. found that dogs from Eastern Europe were more anxious in all fear categories (‘stranger-directed fear’, ‘non-social fear’, ‘dog-directed fear’) and more aggressive towards strangers than dogs from Southern Europe. They discussed that breeds and consequently their typical characteristics vary. For instance, dogs from Southern Europe are predominantly (retired) hunting dogs, whereas dogs from Eastern Europe often display the characteristics of sheepdogs or herding dogs. It is conceivable that dogs from tourist regions (as especially found in Southern Europe) are accustomed to people and therefore adapt more easily to living with humans. There are also many other factors that could play a role, such as climatic conditions or how people interact with dogs.

Regarding the variable ‘type of placement’ (direct vs. indirect via a German animal shelter or foster home), more specific investigations would be advantageous, as we were only able to include relatively few placements through a German animal shelter or foster home in our study. Nevertheless, shelters and foster homes might preselect more challenging dogs, which could explain the (non-significant) slightly lower personality scores.

Regarding the new owner and housing setting, we only found significant positive effects at certain telephone interview times on the personality scores of rescued dogs living with other dogs in the household in Germany (at 6w., 6mos.) and of those that did not undergo external dog training (at 12w., 6mos.). The presence of companion dogs in the household can reduce a dog’s stress level^25,26,35^, which could have led to more desired and relaxed dog behaviour. These results disagree with those of Döring et al^18^., who found that other dogs in the household played no role in the behaviour of adopted laboratory dogs. Our finding regarding external dog training contradicts that of Kubinyi et al^20^., who showed that ‘dogs without any professional training courses were less calm, less trainable and less sociable than trained dogs’. In contrast, Döring et al^19^. also recognised lower personality scores in laboratory dogs with external dog training classes. They discussed that obedience training by their owners might be more important to dogs than external dog training classes and that owners of dogs with behavioural problems would be more likely to seek help and attend dog training^19^. The lower personality scores were therefore likely not the result of external training, but rather the reason why external training was sought^19^. This explanation could also apply to the rescued dogs in our study. In fact, dogs that had received external training in the early phase of adoption (at 1w.) had the lowest mean personality score, suggesting that these dogs exhibited undesirable behaviour at the outset. This could have been amplified in our study because dog training schools were temporarily closed and only owners with a compelling reason persisted in seeking help. The quality of the dog training classes could not be specified in our study.

We found no significant influence of the owner’s experience with dogs on the personality scores. In contrast, other studies have addressed the difference between experienced and inexperienced dog owners. Cohen and Todd^36^presumed that first-time dog owners may not recognise certain behaviours or may not be aware of their impact on everyday life. In addition to the selection of the dog, Mikkola et al^37^. also discussed that experienced dog owners could recognise problems earlier, when they are still easier to treat.

Regarding the type of residential area (rural or urban), we did not identify any significant influence on the dogs’ personality scores. In contrast, other studies have shown that dogs in cities exhibit more fear behaviour than dogs in rural areas^23,24^.

We furthermore compared the desirable and undesirable behaviours of rescued dogs from abroad with the findings of other studies and, in particular, with the findings regarding adopted laboratory dogs^18,19^. We found the incidence of separation problems in rescued dogs (1w.: 44.9% of *n*= 118) as high as in Norman et al^17^. (41.0% of *n*= 3080) and decreasing over months as it did in Demirbas et al^15^. (32.0% to 13.3% of *n* = 75). In contrast, the incidence of separation problems in laboratory dogs^18^ increased over the course of 12 weeks (1w.: 13.0% of *n* = 114, 12w.: 26.0% of *n* = 124). The initially very high incidence of separation problems among rescued dogs could be due to some of the dogs having gone through several changes of ownership and placements. Traumatic separation, relocation to a new home and adoption from shelters are considered risk factors for separation anxiety in dogs^38,39^. The laboratory dogs of Döring et al^19^. as well as the rescued dogs from abroad predominantly displayed desirable behaviour, such as ‘friendly contact’ towards visitors or tolerating situations such as those assessed in the ‘manipulations’ score. The rescued dogs showed ‘fear and avoidance’ behaviour less frequently than the laboratory dogs when confronted with unknown objects and noises (Table 9). Laboratory dogs showed more fear and aggression towards visitors whereas rescued dogs from abroad showed these behaviours more often towards passersby. Compared with the laboratory dogs, the rescued dogs also showed aggression towards their owner in several categories (such as ‘manipulations’, ‘care’, ‘examination’, ‘petting’; Table 9), which rarely occurred in the former laboratory dogs. This could be because purpose-bred laboratory dogs are selected for non-aggressive behaviour. Playing was shown less by rescued dogs than laboratory dogs after the first and twelfth week. Playing is considered an adaptive trait of dogs through domestication and benefits the emotional bond with humans^40^. Dog owners reported that being greeted by their dog and playing with them served as a signal that the dog had settled into their daily routine^41^. More rescued dogs than laboratory dogs took food from their owner’s hand. In an Indian study^42^ conducted over a 15-day period, free-roaming dogs were rewarded either with additional food or social rewards (petting them three times on the head). Social rewards by the experimenter were far more effective than food in building long-term trust^42^. Dogs that received only additional food remained cautious, whereas petted dogs showed a higher ‘socialisation index’ (e.g. wagging their tail towards the experimenter) and increasingly accepted food from the hand. Regarding the willingness to adopt another dog as of week one and week twelve, more dog owners of laboratory dogs than dog owners of rescued dogs in our study stated they would adopt another dog in future (Table 9).

**Table 9:** Comparison of selected results of unwanted behaviours of laboratory dogs according to Döring et al^18,19^. and of the rescued dogs in our study at telephone interview times 1w. and 12w.

Behaviour category	Rescued dogs from this study, 1w.	Laboratory dogs from Döring et al^18,19^., 1w.	Rescued dogs from this study, 12w.	Laboratory dogs from Döring et al^18,19^., 12w.
Separation problems	44.9% (of *n* = 118)	13.0% (of *n* = 114)	26.2% (of *n* = 145)	26.0% (of *n* = 124)
**Unknown noise**				
Subsequent/second reaction, fear and avoidance	28.4% (of *n* = 154)	32.0% (of *n* = 136)	20.5% (of *n* = 150)	20.0% (of *n* = 124)
**Unknown object**				
Subsequent/second reaction, fear and avoidance	26.5% (of *n* = 155)	39.0% (of *n* = 139)	25.2% (of *n* = 151)	39.0% (of *n* = 120)
**Contact with visitor**				
Fear and avoidance	16.7% (of *n* = 126)	27.0% (of *n* = 137)	9.0% (of *n* = 144)	12.0% (of *n* = 124)
Offensive or defensive aggression (mainly involving ‘barking’)	12.6% (of *n* = 126)	5.0% (of *n* = 137)	16.0% (of *n* = 144)	20.0% (of *n* = 124)
**Passerby**				
Fear and avoidance	26.4% (of *n* = 144)	22.0% (of *n* = 138)	20.3% (of *n* = 148)	17.0% (of *n* = 121)
Offensive or defensive aggression	7.0% (of *n* = 144)	3.0% (of *n* = 138)	8.8% (of *n* = 148)	3.0% (of *n* = 121)
**Unknown dog**				
Fear and avoidance	11.6% (of *n* = 138)	12.0% (of *n* = 132)	4.8% (of *n* = 147)	10.0% (of *n* = 124)
Offensive or defensive aggression	23.1% (of *n* = 138)	10.0% (of *n* = 132)	24.5% (of *n* = 147)	9.0% (of *n* = 124)
**Playing**				
Does not play	51.0% (of *n* = 155)	43.0% (of *n* = 143)	34.4% (of *n* = 151)	27.0% (of *n* = 126)
**Feeding out of hand ** **(indoors)**				
Does not eat out of hand	5.8% (of *n* = 155)	12.0% (of *n* = 139)	2.0% (of *n* = 151)	5.0% (of *n* = 126)
**Leaning over the dog (by owner)**				
Moves away	11.9% (of *n* = 151)	6.0% (of *n* = 137)	4.0% (of *n* = 150)	2.0% (of *n* = 126)
Aggression	0.0% (of *n* = 151)	0.0% (of *n =* 137)	0.7% (of *n* = 150)	0.0% (of *n* = 126)
**Carrying the dog (by owner)**				
Moves away	4.3% (of *n* = 138)	1.0% (of *n* = 142)	4.4% (of *n* = 135)	0.0% (of *n* = 124)
Aggression	0.0% (of *n* = 138)	0.0% (of *n* = 142)	0.7% (of *n* = 135)	1.0% (of *n* = 124)
**Taking food away (by owner)**				
Moves away	0.0% (of *n* = 117)	0.0% (of *n* = 109)	1.4% (of *n* = 138)	0.0% (of *n =* 124)
Aggression	5.1% (of *n* = 117)	4.0% (of *n* = 109)	7.2% (of *n* = 138)	6.0% (of *n =* 124)
**Care (brushing the dog by owner)**				
Moves away	4.0% (of *n* = 100)	6.0% (of *n* = 128)	1.5% (of *n* = 136)	2.0% (of *n* = 123)
Aggression	4.0% (of *n* = 100)	0.0% (of *n* =128)	1.5% (of *n* = 136)	0.0% (of *n* = 123)
**Examination (by owner)**				
Moves away	1.4% (of *n* = 144)	2.0% (of *n* = 142)	1.4% (of *n* = 147)	1.0% (of *n* = 126)
Aggression	2.1% (of *n* = 144)	0.0% (of *n* = 142)	1.4% (of *n* = 147)	0.0% (of *n* = 126)
**Willingness to adopt another dog in future**				
Yes (adopt another)	84.5% (of *n* = 155)	90.0% (of *n* = 141)	82.1% (of *n* = 151)	92.0% (of *n* = 123)
No	3.2% (of *n* = 155)	4.0% (of *n* = 141)	6.6% (of *n* = 151)	6.0% (of *n* = 123)
Rescued dogs: only under certain conditions	12.3% (of *n* = 155)	-	11.3% (of *n* = 151)	-
Laboratory dogs: I don’t know	-	1.0% (of *n* = 141)	-	2.0% (of *n* = 123)
Laboratory dogs: only as foster dog	-	5.0% (of *n* = 141)		-

In the case of the two dogs that did not leave the house during the six months of our study, the question arises as to whether they should have been rehomed at all. Reputable animal welfare organisations must assess whether a dog can be accustomed to life in a German household. A dog’s level of socialisation is shaped by the experiences it has between the ages of three and twenty weeks^43^. If this phase is missed or has a negative impact, the dog can still be accustomed to people later on, but building trust becomes significantly more difficult and takes longer^43^. According to Hirt et al^44^., severe anxiety can be regarded as suffering as defined in the German Animal Welfare Act. If such a situation is reported to a veterinary authority in Germany, conditions may be imposed, such as the involvement of a veterinarian specialising in behaviour.

To our knowledge, this study is the first prospective investigation of the behaviour of rescued dogs from abroad after they have been placed in private households. This study has both strengths and limitations. It is advantageous to ask owners about the current period of their dog’s behaviour because memories can become blurred and owners can give circumstances more or less importance (recall bias)^45^. That is why prospective studies are recommended over retrospective studies. Recall bias can also occur in prospective studies, but it can be prevented by certain methods, such as standardised questionnaires like the ones we used in this study^46^. The specific questioning of defined behavioural categories instead of subjective impressions is also an advantage, even if a behavioural diagnosis cannot be made on the basis of this questionnaire. Although the personality score was based on 18 objectified behavioural scales, it is important to bear in mind that these are the dog owner’s assessments, not those of experts. The participants were highly motivated from the outset, which could be considered a limitation and could have resulted in selection bias. This may explain why there were no significant differences between experienced owners and first-time owners: all participants were highly motivated and may have been already informed about rescued dogs from abroad, for example how to recognise signs of distress or illnesses. However, it is precisely this sampling error of motivated participants that can be useful because they are very interested and committed and make very accurate assessments^47^. Problematic in telephone interviews compared with online surveys is the high influence of social desirability^48^. Especially on the telephone, respondents tend to give answers that they believe are more in line with the expectations of researchers and society^49^. Online surveys, on the other hand, only reach people who are tech-savvy. Unlike previous studies, which relied solely on online surveys, we were able to include people outside the digital world in our study through telephone interviews.

There are some limitations arising from the data situation and the applied tests. The observed dogs cannot be considered as independent and identically distributed. Some owners adopted more than one dog, and we included all adopted dogs in our analysis. Also, dogs can share a similar history when they stem from the same organisation. This may affect the robustness of the estimated *p*-values.

To facilitate a comparison, our methodology was based on the laboratory dog studies by Döring et al^18,19^. It would be desirable to repeat the study by using German shelter dogs and German pedigree dogs in order to conduct a comparative analysis of the differences in behavioural adaptation and development of the dogs with their new owners, as well as of any behavioural problems that may arise.

## Conclusions

Our results showed that most of the dogs we examined were suitable as pets. The dogs changed their behaviour after their adoption, at least during the first six months. Behavioural development was significantly positive, but it did not progress in a linear manner. Adopters of dogs from abroad should expect that the dogs may exhibit separation problems and fear of unknown objects and noises. Likewise, some dogs showed fear or aggression towards the owner in certain situations, towards visitors and towards passersby, particularly towards men. As we found no significant influence of the dogs’ sex, neutering status, size and age in our study, no recommendation can be made for a specific age or sex group.

Information about the dog’s behaviour and health provided by animal welfare organisations should be critically questioned because owners mentioned discrepancies between the information provided and their own experiences. There were individual dogs with long-term suffering, for example two dogs that were unable to leave the house over several months owing to intense anxiety, and dogs obviously suffering from severe distress in multiple situations. Some of these dogs should not have been imported.

Our study dogs seemed to settle faster with other dogs in the household. Therefore, we conclude that other dogs in the household can have a positive effect on a rescued dog’s behaviour. Not all rescued dogs played or took food from their owner’s hand. This finding should be considered when training dogs. Rescued dogs should not be expected to be ‘suitable for everyday life’ from the outset. Instead, owners should have realistic expectations and patience with their adopted dogs.

## Supplementary Information

Below is the link to the electronic supplementary material.

Supplementary Material 1

## Data Availability

The data is available from the corresponding author on reasonable request.

## References

[1] Hughes, J. & Macdonald, D. W. A review of the interactions between free-roaming domestic dogs and wildlife. *Biol. Conserv.* **157**, 341–351 (2013).

[2] Papavasili, T. et al. Review of stray dog management: dog days in European countries*.**Bulgarian J. Veterinary Med. ***27 **(2) (2024).

[3] ICAM, *Humane Dog Population Guidance* (2019). https://www.icam-coalition.org/download/humane-dog-population-management-guidance/.

[4] World Organisation for Animal Health (WOAH), *Terrestrial Animal Health Code: Chap. 7.7. Stray dog population control* (2018). https://www.woah.org/fileadmin/Home/eng/Health_standards/tahc/2018/en_chapitre_aw_stray_dog.htm.

[5] Smith, L. M. et al. The effectiveness of dog population management: A systematic review. *Animals* **9** (12), 1020 (2019). 31766746 10.3390/ani9121020 PMC6940938

[6] Warembourg, C. et al. Comparative Study of Free-Roaming Domestic Dog Management and Roaming Behavior Across Four Countries: Chad, Guatemala, Indonesia, and Uganda. *Front. Veterinary Sci. *Volume **8 ** (2021). 10.3389/fvets.2021.617900 PMC7970034 33748208

[7] Deutscher Tierschutzbund. *Illegaler Heimtierhandel und seine Auswirkungen auf deutsche Tierheime – Auswertung bekannt gewordener Fälle aus dem Jahr 2022 *(2023). www.tierschutzbund.de.

[8] Naucke, T. J. et al. Prevalence of canine vector-borne pathogens in imported and travelling dogs in Germany and studies on the prevention of CVBD.* Tierärztliche Umschau* **66**, 311–317 (2011).

[9] Hamel, D., Röhrig, E. & Pfister, K. Canine vector-borne disease in travelled dogs in Germany—A retrospective evaluation of laboratory data from the years 2004–2008. *Vet. Parasitol.* **181** (1), 31–36 (2011). 21565447 10.1016/j.vetpar.2011.04.020

[10] Loeb, J. & Gray, A. Compassion v biosecurity: are dog rescues driving disease emergence? *Wiley Online Library, *192–193 (2022). 10.1002/vetr.2192 36083077

[11] Schäfer, I. et al. Retrospective evaluation of vector-borne infections in dogs imported from the Mediterranean region and southeastern Europe (2007–2015). *Parasites & Vectors* Vol. **12**, 30 (2019). 10.1186/s13071-018-3284-8 PMC6330426 30635034

[12] Tierärztliche Vereinigung für Tierschutz (TVT), Hunde- und Katzenimporte- Merkblatt 113 (2023). https://www.tierschutz-tvt.de/alle-merkblaetter-und-stellungnahmen/.

[13] Buckley, L. A. Imported rescue dogs: lack of research impedes evidence-based advice to ensure the welfare of individual dogs. *Vet. Rec.* **186** (8), 245 (2020). 32108063 10.1136/vr.m653

[14] Graf, J., Kuhne, F. & Serpell, J. A. Behavioral traits of rescue dogs from Southern and Eastern Europe rehomed to Germany. *J. Veterinary Behav.* **77**, 77–85 (2025).

[15] Demirbas, Y. S., Emre, B. & Kockaya, M. Integration ability of urban free-ranging dogs into adoptive families’ environment. *J. Vet. Behav.* **9 **(5), 222–227 (2014).

[16] Munkeboe, N. et al. Comparing behavioural problems in imported street dogs and domestically reared danish dogs—the views of dog owners and veterinarians. *Animals* **11** (5), 1436 (2021). 34067927 10.3390/ani11051436 PMC8157144

[17] Norman, C., Stavisky, J. & Westgarth, C. Importing rescue dogs into the UK: reasons, methods and welfare considerations. *Vet. Rec.* **186** (8), 248–248 (2020). 31932354 10.1136/vr.105380 PMC7057815

[18] Döring, D. et al. Behavior of laboratory dogs before and after rehoming in private homes. *ALTEX-Alternatives Anim. Experimentation*. **34** (1), 133–147 (2017). 10.14573/altex.1608171 27725989

[19] Döring, D. et al. How do rehomed laboratory beagles behave in everyday situations? Results from an observational test and a survey of new owners. *PloS One*. **12** (7), e0181303 (2017). 28742824 10.1371/journal.pone.0181303 PMC5526562

[20] Kubinyi, E., Turcsán, B. & Miklósi, Á. Dog and owner demographic characteristics and dog personality trait associations. *Behav. Process.* **81** (3), 392–401 (2009). 10.1016/j.beproc.2009.04.004 19520239

[21] McGreevy, P. D. et al. Dog behavior co-varies with height, bodyweight and skull shape. *PloS One*. **8** (12), e80529 (2013). 24358107 10.1371/journal.pone.0080529 PMC3864788

[22] Martínez, Á. G. et al. Risk factors associated with behavioral problems in dogs. *J. Vet. Behav.* **6 **(4), 225–231 (2011).

[23] Hakanen, E. et al. Active and social life is associated with lower non-social fearfulness in pet dogs. *Sci. Rep.* **10 **(1), 13774 (2020). 32792641 10.1038/s41598-020-70722-7 PMC7426946

[24] Puurunen, J. et al. Inadequate socialisation, inactivity, and urban living environment are associated with social fearfulness in pet dogs. *Sci. Rep.* **10 **(1), 3527 (2020). 10.1038/s41598-020-60546-w PMC7044223 32103117

[25] Mariti, C. et al. Intraspecific attachment in adult domestic dogs (Canis familiaris): Preliminary results. *Appl. Anim. Behav. Sci.* **152**, 64–72 (2014).

[26] Dreschel, N. A. & Granger, D. A. Physiological and behavioral reactivity to stress in thunderstorm-phobic dogs and their caregivers. *Appl. Anim. Behav. Sci.* **95** (3–4), 153–168 (2005).

[27] Gates, M. C. et al. Post-adoption problem behaviours in adolescent and adult dogs rehomed through a New Zealand animal shelter. *Animals* **8** (6), 93 (2018). 29891756 10.3390/ani8060093 PMC6024916

[28] Diesel, G., Pfeiffer, D. & Brodbelt, D. Factors affecting the success of rehoming dogs in the UK during 2005. *Prev. Vet. Med.* **84** (3–4), 228–241 (2008). 18243374 10.1016/j.prevetmed.2007.12.004

[29] Blackwell, E. J., Casey, R. A. & Bradshaw, J. W. Efficacy of written behavioral advice for separation-related behavior problems in dogs newly adopted from a rehoming center. *J. Veterinary Behav.* **12**, 13–19 (2016).

[30] Poulsen, A., Lisle, A. & Phillips, C. An evaluation of a behaviour assessment to determine the suitability of shelter dogs for rehoming. *Veterinary Med. Int.* **2010** (1), 523781 (2010). 10.4061/2010/523781 PMC2859023 20445786

[31] Landis, J. R. & Koch, G. G. The measurement of observer agreement for categorical data. *Biometrics* **33** (1), 159–174 (1977). 843571

[32] Corsetti, S., Pimpolari, L. & Natoli, E. How different personalities affect the reaction to adoption of dogs adopted from a shelter. *Animals* **11** (6), 1816 (2021). 34207105 10.3390/ani11061816 PMC8234085

[33] Starling, M. J. et al. Age, sex and reproductive status affect boldness in dogs. *Vet. J.* **197 **(3), 868–872 (2013). 23778256 10.1016/j.tvjl.2013.05.019

[34] Scandurra, A. et al. Behavioral and perceptual differences between sexes in dogs: An overview. *Animals* **8** (9), 151 (2018). 30142932 10.3390/ani8090151 PMC6162565

[35] Sipple, N. et al. *Integrative and Comparative Biology*. Vol. 61. 132–139. (2021). 10.1093/icb/icab054 33970264

[36] Cohen, S. E. & Todd, P. M. Stated and revealed preferences in companion animal choice. *Behav. Res. Methods* **51 **(4), 1498–1509 (2019). 31065937 10.3758/s13428-019-01253-x

[37] Mikkola, S. et al. Aggressive behaviour is affected by demographic, environmental and behavioural factors in purebred dogs. *Sci. Rep.* **11 **(1), 9433 (2021). 33941802 10.1038/s41598-021-88793-5 PMC8093277

[38] Serpell, J. Early experience and the development of behaviour, in *The domestic dog,* 80–102 (1995).

[39] Schwartz, S. Separation anxiety syndrome in dogs and cats. *J. Am. Vet. Med. Assoc.* **222** (11), 1526–1532 (2003). 12784957 10.2460/javma.2003.222.1526

[40] Bradshaw, J. W., Pullen, A. J. & Rooney, N. J. Why do adult dogs ‘play’? *Behav. Processes* **110**, 82–87 (2015). 25251020 10.1016/j.beproc.2014.09.023

[41] Moyer, B. J. et al. A qualitative exploration of owner experiences following dog adoption. *Anim. Welf.* **34**, e9. (2025). 10.1017/awf.2025.4 PMC11810508 39935773

[42] Bhattacharjee, D. et al. Free-ranging dogs prefer petting over food in repeated interactions with unfamiliar humans. *J. Exp. Biol.* **220** (24), 4654–4660 (2017). 29038310 10.1242/jeb.166371

[43] Feddersen-Petersen, D. *Hundepsychologie: Sozialverhalten und Wesen-Emotionen und Individualität* 237–307 (Kosmos, 2004).

[44] Hirt, A., Maisack, C. & Moritz, J. *Tierschutzgesetz: TierSchG* 19–26 (p. Rn, 2016).

[45] Talari, K. & Goyal, M. Retrospective studies–utility and caveats. *J. Royal Coll. Physicians Edinb.* **50** (4), 398–402 (2020). 10.4997/JRCPE.2020.409 33469615

[46] Hassan, E. Recall bias can be a threat to retrospective and prospective research designs. *Internet J. Epidemiol.* **3** (2), 339–412 (2006).

[47] Hofmann, N. *Aktueller Stand der tierärztlichen Forschung zu privater Tierhaltung mittels Online-Befragungen* (Ludwig-Maximilians-Universität, 2019).

[48] Zhang, X. et al. Survey method matters: Online/offline questionnaires and face-to-face or telephone interviews differ. *Computers Hum. Behav. Rep.* **71**, 172–180 (2017).

[49] Grimm, P. Social desirability bias. In *Wiley international encyclopedia of marketing* (2010).

