# Supplementary material for: Behavioural adaptation of 158 rescued dogs from abroad during the first six months after adoption in Germany
Source: Sci Rep. 2026 Jul 30;16:23613. doi: 10.1038/s41598-026-62598-w (PMC13424329; doi:10.1038/s41598-026-62598-w)
Supplement: Supplementary file 1 — Supplementary Material 1 [file 41598_2026_62598_MOESM1_ESM.docx]

Supplementary Information

**Table S1. Parameters, behaviour categories, behaviour scales and results according to information provided by the owners at the time of the telephone interviews from week one to month six.**

| **Parameter and Categories** | **Description or question asked** | | **Scale** | | **1w.  *n*=154** | | **6w. *n*=151** | | **12w.  *n*=151** | | **6mos. *n*=149** | |
| --- | --- | --- | --- | --- | --- | --- | --- | --- | --- | --- | --- | --- |
|  |  |  | | | No. | % | No. | % | No. | % | No. | % |
| **Isolation** |  | | |  | *n*=118 | | *n*=142 | | *n*=145 | | *n*=146 | |
| calm | dog does not bark, howl or whine and does not destroy anything | | | **3** | 65 | 55.1% | 104 | 73.2% | 107 | 73.8% | 111 | 76.0% |
| separation problems | dog barks, howls or whines more than 3 min and/or destroys objects in the home | | | **0** | 53 | 44.9% | 38 | 26.8% | 38 | 26.2% | 35 | 23.9% |
| **Contact with visitor** |  | | |  | *n*=126 | | *n*=136 | | *n*=144 | | *n*=144 | |
| How does the dog behave towards visitors in your house/apartment? | | | |  |  | |  | |  | |  | |
| friendly contact | dog walks toward the person in a speedy manner with a relaxed body posture and licks/ sniffs/ jumps up | | | **3** | 57 | 45.2% | 60 | 44.1% | 74 | 51.4% | 76 | 52.8% |
| cautious contact | dog hesitantly approaches the person with signals of fear, doesn`t jump up, watches person/ sniffs/ licks | | | **2** | 23 | 18.3% | 31 | 22.8% | 19 | 13.2% | 18 | 12.5% |
| fear and avoidance | dog does not approach the person; dog moves away when the person approaches him/her and shows signals of fear e.g. crouched posture, tucked tail | | | **0** | 21 | 16.7% | 13 | 9.6% | 13 | 9.0% | 13 | 9.0% |
| does something else | dog does not seek contact because he is busy doing something else e.g. stays in its place (no change of current behaviour) | | | **2** | 9 | 7.1% | 11 | 8.1% | 15 | 10.4% | 9 | 6.3% |
| offensive aggression | dog approaches the person and bares teeth or barks or growls or snaps or bites | | | **0** | 6 | 4.7% | 14 | 10.3% | 15 | 10.4% | 22 | 15.3% |
|  | baring teeth | | |  | 1 | 0.8% | 0 | 0.0% | 1 | 0,7% | 0 | 0.0% |
|  | barking | | |  | 6 | 4.7% | 11 | 8.1% | 13 | 9.0% | 19 | 13.2% |
|  | growling | | |  | 4 | 3.2% | 5 | 3.7% | 2 | 1.4% | 6 | 4.2% |
|  | snapping | | |  | 0 | 0.0% | 0 | 0.0% | 0 | 0.0% | 1 | 0.7% |
|  | biting | | |  | 1 | 0.8% | 0 | 0.0% | 0 | 0.0% | 0 | 0.0% |
| defensive aggression | dog bares teeth or barks or growls or snaps or bites when being approached by the person | | | **0** | 10 | 7.9% | 7 | 5.1% | 8 | 5.6% | 6 | 4.2% |
|  | baring teeth | | |  | 0 | 0.0% | 1 | 0,7% | 1 | 0.7% | 0 | 0.0% |
|  | barking | | |  | 8 | 6.3% | 4 | 2.9% | 4 | 2.8% | 5 | 3.5% |
|  | growling | | |  | 5 | 4.0% | 5 | 3.7% | 4 | 2.8% | 0 | 0.0% |
|  | snapping | | |  | 0 | 0.0% | 0 | 0.0% | 0 | 0.0% | 1 | 0.7% |
|  | biting | | |  | 0 | 0.0% | 0 | 0.0% | 0 | 0.0% | 0 | 0.0% |
| Is there a difference between men, women, children, older people or people in wheelchairs? | | | |  | *n*=126 | | *n*=136 | | *n*=144 | | *n*=144 |  |
|  | no | | |  | 90 | 71.4% | 95 | 69.9% | 100 | 69.4% | 106 | 73.6% |
|  | yes | | |  | 36 | 28.5% | 41 | 30.1% | 44 | 30.6% | 38 | 26.4% |
|  | more hesitant/anxious towards men | | |  | 24 | 19.0% | 23 | 16.9% | 25 | 17.4% | 21 | 14.5% |
|  | more aggressive towards men | | |  | 2 | 1.6% | 1 | 0.1% | 1 | 0.7% | 2 | 1.4% |
|  | men more popular | | |  | 2 | 1.6% | 0 | 0.0% | 1 | 0.7% | 2 | 1.4% |
|  | more anxious towards children | | |  | 6 | 4.8% | 10 | 7.4% | 11 | 7.6% | 11 | 7.6% |
|  | children more popular | | |  | 5 | 4.0% | 9 | 6.6% | 9 | 6.3% | 4 | 2.8% |
|  | women more popular | | |  | 3 | 2.4% | 6 | 4.4% | 5 | 3.5% | 3 | 2.1% |
|  | more anxious towards women | | |  | 0 | 0.0% | 0 | 0.0% | 2 | 1.4% | 0 | 0.0% |
|  | anxious towards wheelchair users | | |  | 0 | 0.0% | 2 | 1.5% | 1 | 0.7% | 1 | 0.7% |
| **Passerby** |  | | |  | *n*=144 | | *n*=146 | | *n*=148 | | *n*=146 | |
| How does the dog behave when you meet a stranger outside? | | | |  |  | |  | |  | |  | |
| friendly contact | dog walks toward the person in a speedy manner with a relaxed body posture and licks/ sniffs/ jumps up | | | **3** | 46 | 31.9% | 50 | 34.2.% | 37 | 25.0% | 44 | 30.1% |
| cautious contact | dog hesitantly approaches the person with signals of fear, doesn`t jump up, watches person/ sniffs/ licks | | | **2** | 19 | 13.2% | 19 | 13.0% | 15 | 10.1% | 15 | 10.3% |
| fear and avoidance | dog does not approach the person, dog moves away when being approached by the person and shows signals of fear e.g. crouched posture, tucked tail | | | **0** | 38 | 26.4% | 30 | 20.5% | 30 | 20.3% | 20 | 13.7% |
| does something else | dog does not seek contact because he is busy doing something else e.g. stays in its place (no change of current behaviour) | | | **3** | 31 | 21.5% | 35 | 24.0% | 53 | 35.8% | 54 | 37.0% |
| offensive aggression | dog approaches the person and bares teeth or barks or growls or snaps or bites | | | **0** | 7 | 4.9% | 7 | 4.8% | 9 | 6.1% | 9 | 6.2% |
|  | baring teeth | | |  | 0 | 0.0% | 0 | 0.0% | 1 | 0.7% | 2 | 1.4% |
|  | barking | | |  | 6 | 4.2% | 6 | 4.1% | 9 | 6.1% | 7 | 4.8% |
|  | growling | | |  | 3 | 2.1% | 2 | 1.4% | 3 | 2.0% | 4 | 2.7% |
|  | snapping | | |  | 0 | 0.0% | 0 | 0.0% | 0 | 0.0% | 1 | 0.7% |
|  | biting | | |  | 0 | 0.0% | 0 | 0.0% | 0 | 0.0% | 0 | 0.0% |
| defensive aggression | dog bares teeth or barks or growls or snaps or bites when being approached by the person | | | **0** | 3 | 2.1% | 5 | 3.4% | 4 | 2.7% | 4 | 2.7% |
|  | baring teeth | | |  | 0 | 0.0% | 0 | 0.0% | 0 | 0.0% | 0 | 0.0% |
|  | barking | | |  | 3 | 2.1% | 5 | 3.4% | 4 | 2.7% | 4 | 2.7% |
|  | growling | | |  | 1 | 0.7% | 2 | 1.4% | 2 | 1.4% | 1 | 0.7% |
|  | snapping | | |  | 0 | 0.0% | 0 | 0.0% | 0 | 0.0% | 0 | 0.0% |
|  | biting | | |  | 0 | 0.0% | 0 | 0.0% | 0 | 0.0% | 0 | 0.0% |
| Is there a difference between men, women, children, older people or people in wheelchairs? | | | |  | *n*=140 | | *n*=146 | | *n=*148 | | *n=*146 |  |
|  | no | | |  | 91 | 65.0% | 97 | 66.4% | 98 | 66.2% | 107 | 73.3% |
|  | yes | | |  | 49 | 35.0% | 49 | 33.6% | 50 | 33.8% | 39 | 26.7% |
|  | more hesitant/anxious towards men | | |  | 29 | 20.7% | 23 | 15.8% | 19 | 12.8% | 17 | 11.6% |
|  | more aggressive towards men | | |  | 2 | 1.4% | 1 | 0.7% | 0 | 0.0% | 3 | 2.1% |
|  | men more popular | | |  | 2 | 1.4% | 0 | 0.0% | 0 | 0.0% | 1 | 0.7% |
|  | more anxious towards children | | |  | 6 | 4.2% | 9 | 6.2% | 7 | 4.7% | 3 | 2.1% |
|  | children more popular | | |  | 7 | 5.0% | 8 | 5.5% | 4 | 2.7% | 3 | 2.1% |
|  | women more popular | | |  | 3 | 2.1% | 4 | 2.7 % | 2 | 1.4 % | 0 | 0.0% |
|  | more anxious towards women | | |  | 2 | 1.4% | 0 | 0.0% | 2 | 1.4% | 1 | 0.7% |
|  | people with hats or similar | | |  | 4 | 2.8 % | 0 | 0.0 % | 4 | 2.7 % | 0 | 0.0 % |
|  | people with rollators, baby carriages, etc. | | |  | 7 | 5.0 % | 4 | 2.7 % | 7 | 4.7 % | 2 | 1.4 % |
|  | different skin colour or conspicuous clothing colour | | |  | 0 | 0.0 % | 1 | 0.7 % | 1 | 0.6 % | 0 | 0.0 % |
| **Reaction to unknown object** | Behaviour toward unknown objects (e.g., trash can, vacuum cleaner, suitcase, broom): Have you noticed that your dog is afraid or uncomfortable when it sees certain objects? | | |  | *n*=155 | | *n*=151 | | *n*=151 | | *n*=149 | |

| No |  |  | 65 | 41.9% | 74 | 49.0% | 80 | 53.0% | 79 | 53.0% |
| --- | --- | --- | --- | --- | --- | --- | --- | --- | --- | --- |
| Yes |  |  | 90 | 58.1% | 77 | 51.0% | 71 | 47.0% | 70 | 47.0% |
|  | These are the objects: |  |  |  |  |  |  |  |  |  |
|  | Vacuum cleaner |  | 53 | 34.2% | 26 | 17.2% | 29 | 19.2% | 14 | 9.4% |
|  | Broom |  | 9 | 5.9% | 5 | 3.3% | 3 | 2.0% | 8 | 5.4% |
|  | Trash can |  | 8 | 5.1% | 10 | 6.6% | 5 | 3.3% | 8 | 5.4% |
|  | Tarpaulin, bag |  | 5 | 3.2% | 3 | 2.0% | 6 | 4.0% | 0 | 0.0% |
|  | Household and garden appliances |  | 8 | 5.1% | 3 | 2.0% | 5 | 3.3% | 5 | 3.4% |
|  | Figurines, statues |  | 4 | 2.6% | 2 | 1.3% | 1 | 0.6% | 5 | 3.4% |
|  | Furniture |  | 4 | 2.6% | 0 | 0.0% | 0 | 0.0% | 1 | 0.7% |
|  | Sticks |  | 4 | 2.6% | 1 | 0.6% | 6 | 4.0% | 4 | 2.7% |
|  | Strollers |  | 4 | 2.6% | 3 | 2.0% | 1 | 0.6% | 0 | 0.0% |
|  | Other |  | 6 | 3.9% | 4 | 2.6% | 6 | 4.0% | 22 | 14.8% |

| How does your dog react? |  |  |  | |  | |  | |  | |
| --- | --- | --- | --- | --- | --- | --- | --- | --- | --- | --- |
| **First Reaction** |  |  |  |  |  |  |  |  |  |  |
| gets frightened | dog flinches and backs away, showing fear behaviour such as trembling, crawling or tucked tail for longer than 30 seconds | **0** | 38 | 24.5% | 29 | 19.2% | 26 | 17.2 % | 12 | 8.1% |
| gets startled | dog flinches and backs away, showing fear behaviour such as trembling, crawling or tucked tail for less than 30 seconds | **1** | 57 | 36.8% | 50 | 33.1% | 45 | 29.8 % | 62 | 41.6% |
| is relaxed | dog does not flinch or move back | **3** | 60 | 38.7% | 72 | 47.7% | 80 | 53.0 % | 75 | 50.3% |
| **Subsequent Reaction** |  |  |  |  |  |  |  |  |  |  |
| makes contact | dog approaches and watches object and/or tries to make contact with his/her snout | **3** | 66 | 42.6% | 72 | 47.7% | 68 | 45.0 % | 74 | 49.7% |
| fear and avoidance | dog moves back and/or stays at a distance, shows signals of fear e.g. trembling, crawling or tucked tail | **0** | 41 | 26.5% | 36 | 23.8% | 38 | 25,2 % | 33 | 22.1% |
| is relaxed | dog shows no reaction, is relaxed and continues its original behaviour | **3** | 48 | 31.0% | 43 | 28.5% | 45 | 29.8 % | 42 | 28.2% |

|  | How long does it take for him to calm down? |  | *n*=90 | | *n*=77 | | *n*=71 | | *n*=70 |  |
| --- | --- | --- | --- | --- | --- | --- | --- | --- | --- | --- |
|  | <1 minute |  | 49 | 54.4% | 43 | 55.8% | 28 | 39.4% | 28 | 40.0% |
|  | 1-2 minutes |  | 21 | 23.3% | 8 | 10.4% | 20 | 28.2% | 13 | 18.6% |
|  | 2-5 minutes |  | 7 | 7.7% | 3 | 3.9% | 2 | 2.8% | 11 | 15.7% |
|  | longer than 5 minutes |  | 5 | 5.5% | 4 | 5.2% | 1 | 1.4% | 5 | 7.1% |
|  | Only when the source is removed/turned off |  | 8 | 8.8% | 12 | 15.6% | 13 | 18.3% | 13 | 18.6% |
|  | No information provided |  | 0 | 0.0% | 7 | 9.1% | 8 | 11.3% | 0 | 0.0% |

| **Reaction to unknown noise** | Behaviour toward unknown noises (e.g., New Year's Eve fireworks, loud bangs, fire engine sirens): Have you noticed that your dog is afraid or uncomfortable when hearing certain noises? |  | *n*=154 | | *n*=150 | | *n*=150 | | *n*=148 | |
| --- | --- | --- | --- | --- | --- | --- | --- | --- | --- | --- |
| No |  |  | 62 | 40.2% | 75 | 49.7% | 79 | 52.3% | 83 | 55.7% |
| Yes |  |  | 92 | 59.7% | 76 | 50.3% | 72 | 47.7% | 66 | 44.3% |
|  | These are the noises: |  |  |  |  |  |  |  |  |  |
|  | Loud bangs, gunshots, New Year's Eve fireworks |  | 25 | 16.2% | 12 | 8.0% | 17 | 11.3% | 31 | 20.9% |
|  | Road traffic |  | 15 | 9.7% | 20 | 13.3% | 14 | 9.3% | 12 | 8.1% |
|  | Sirens |  | 15 | 9.7% | 5 | 3.3% | 2 | 1.3% | 0 | 0.0% |
|  | Hair dryers |  | 10 | 6.5% | 2 | 1.3% | 3 | 2.0% | 0 | 0.0% |
|  | Televisions, radios |  | 9 | 5.8% | 0 | 0.0% | 0 | 0.0% | 0 | 0.0% |
|  | People talking |  | 6 | 4.0% | 0 | 0.0% | 0 | 0.0% | 1 | 0.7% |
|  | Kitchen/garden/construction equipment |  | 6 | 4.0% | 9 | 6.0% | 4 | 2.7% | 3 | 2.0% |
|  | Bells, Doorbells |  | 5 | 3.2% | 1 | 0.6% | 0 | 0.0% | 0 | 0.0% |
|  | Wind, thunder |  | 2 | 1.3% | 3 | 2.0% | 2 | 1.3% | 1 | 0.7% |
|  | Airplanes |  | 2 | 1.3% | 0 | 0.0% | 3 | 2.0% | 0 | 0.0% |
|  | Other |  | 12 | 7.8% | 7 | 4.7% | 4 | 2.7% | 1 | 0.7% |
| How does your dog react? |  |  |  | |  | |  | |  | |
| **First Reaction** |  |  |  |  |  |  |  |  |  |  |
| gets frightened | dog flinches and backs away, showing fear behaviour such as trembling, crawling or tucked tail for longer than 30 seconds | **0** | 35 | 23.2% | 28 | 18.5% | 20 | 13.2% | 25 | 16.8% |
| gets startled | dog flinches and backs away, showing fear behaviour such as trembling, crawling or tucked tail for less than 30 seconds | **1** | 59 | 38.1% | 52 | 34.4% | 52 | 34.4% | 49 | 32.9% |
| is relaxed | dog does not flinch or move back | **3** | 60 | 39.0% | 70 | 46.6% | 78 | 52.3% | 74 | 50.0% |
| **Subsequent Reaction** |  |  |  |  |  |  |  |  |  |  |
| makes contact | dog approaches and watches object and/or tries to make contact with his/her snout | **3** | 48 | 31.0% | 46 | 30.5% | 45 | 29.8% | 39 | 26.2% |
| fear and avoidance | dog moves back and/or stays at a distance, shows signals of fear e.g. trembling, crawling or tucked tail | **0** | 44 | 28.4% | 34 | 22.5% | 31 | 20.5% | 35 | 23.5% |
| is relaxed | dog shows no reaction, is relaxed and continues its original behaviour | **3** | 62 | 40.2% | 70 | 46.6% | 74 | 49.7% | 74 | 50.0% |

|  | How long does it take for him to calm down? |  | *n*=90 | | *n*=80 | | *n*=72 | | *n*=74 |  |
| --- | --- | --- | --- | --- | --- | --- | --- | --- | --- | --- |
|  | <1 minute |  | 45 | 50.0% | 48 | 60.0% | 37 | 51.4% | 37 | 50.0% |
|  | 1-2 minutes |  | 27 | 30.0% | 12 | 15.0% | 15 | 20.8% | 11 | 14.9% |
|  | 2-5 minutes |  | 9 | 10.0% | 3 | 3.8% | 9 | 12.5% | 8 | 10.8% |
|  | longer than 5 minutes |  | 7 | 7.8% | 4 | 5.0% | 3 | 4.2% | 5 | 6.8% |
|  | Only when the source is removed/turned off |  | 4 | 4.4% | 5 | 6.3% | 2 | 2.8% | 3 | 4.1% |
|  | No information provided |  | 0 | 0.0% | 8 | 10.0% | 6 | 8.3% | 10 | 13.5% |

| **Manipulations** |  | | |  | |  |  |  |  |  |  |  |  |
| --- | --- | --- | --- | --- | --- | --- | --- | --- | --- | --- | --- | --- | --- |
| These reactions are only of interest to us if you have already experienced them in everyday life. You should not provoke these situations because they are dangerous: How does your dog behave when you | | | |  | |  |  |  |  |  |  |  |  |
| **leaning over him?** | | | |  | | *n*=151 | | *n*=150 | | *n*=150 | | *n*=147 | |
| acceptance | dog endures/tolerates the situation. |  |  | **3** |  | 118 | 78.1% | 131 | 87.3% | 134 | 89.3% | 134 | 91.2% |
| slight withdrawal | dog tries to escape (head movements or movement of the body). | | | **2** |  | 11 | 7.3% | 6 | 4.0% | 7 | 4.7% | 5 | 3.4% |
| freeing | dog gets out of the situation. |  |  | **1** |  | 4 | 2.7% | 5 | 3.3% | 2 | 1.3% | 2 | 1.4% |
| moves away | dog backs away from me so that the measure cannot be carried out. | | | **0** |  | 18 | 11.9% | 5 | 3.3% | 6 | 4.0% | 5 | 3.4% |
| aggression | dog shows signs of threatening and/or attacking behaviour such as | | | **0** |  | 0 | 0.0% | 3 | 2.0% | 1 | 0.7% | 1 | 0.7% |
|  | baring teeth | | |  |  | 0 | 0.0% | 1 | 0.7% | 0 | 0.0% | 0 | 0.0% |
|  | barking | | |  |  | 0 | 0.0% | 0 | 0.0% | 0 | 0.0% | 0 | 0.0% |
|  | growling | | |  |  | 0 | 0.0% | 1 | 0.7% | 1 | 0.7% | 1 | 0.7% |
|  | snapping | | |  |  | 0 | 0.0% | 0 | 0.0% | 0 | 0.0% | 0 | 0.0% |
|  | biting | | |  |  | 0 | 0.0% | 0 | 0.0% | 0 | 0.0% | 0 | 0.0% |
| **carrying him?** | | | |  | | *n*=138 | | *n*=136 | | *n*=135 | | *n*=133 | |
| acceptance | dog endures/tolerates the situation. |  |  | **3** |  | 114 | 82.6% | 115 | 84.6% | 118 | 87.4% | 109 | 82.0% |
| slight withdrawal | dog tries to escape (head movements or movement of the body). | | | **2** |  | 13 | 9.4% | 10 | 7.4% | 6 | 4.4% | 11 | 8.3% |
| freeing | dog gets out of the situation. |  |  | **1** |  | 5 | 3.6% | 2 | 1.5% | 4 | 3.0% | 2 | 1.5% |
| moves away | dog backs away from me so that the measure cannot be carried out. | | | **0** |  | 6 | 4.3% | 5 | 3.7% | 6 | 4.4% | 8 | 6.0% |
| aggression | dog shows signs of threatening and/or attacking behaviour such as baring teeth, barking, growling, snapping, biting . | | | **0** |  | 0 | 0.0% | 4 | 2.9% | 1 | 0.7% | 3 | 2.3% |
|  | baring teeth | | |  |  | 0 | 0.0% | 1 | 0.7% | 0 | 0.0% | 2 | 1.5% |
|  | barking | | |  |  | 0 | 0.0% | 0 | 0.0% | 0 | 0.0% | 0 | 0.0% |
|  | growling | | |  |  | 0 | 0.0% | 0 | 0.0% | 1 | 0.7% | 1 | 0.8% |
|  | snapping | | |  |  | 0 | 0.0% | 0 | 0.0% | 0 | 0.0% | 1 | 0.8% |
|  | biting | | |  |  | 0 | 0.0% | 0 | 0.0% | 0 | 0.0% | 0 | 0.0% |
| **taking food away?** | | | |  | | *n*=117 | | *n*=130 | | *n*=138 | | *n*=141 | |
| acceptance | dog endures/tolerates the situation. |  |  | **3** | | 111 | 94.9 % | 118 | 90.8% | 126 | 91.3% | 128 | 90.8% |
| moves away | dog moves away from owner. | | | **2** | | 0 | 0.0% | 2 | 1.5% | 2 | 1.4% | 3 | 2.1% |
| aggression | dog shows signs of threatening and/or attacking behaviour such as baring teeth, barking, growling, snapping, biting . | | | **0** | | 6 | 5.1% | 10 | 7.7% | 10 | 7.2% | 10 | 7.1% |
|  | baring teeth | | |  | | 2 | 1.7% | 3 | 2.3% | 3 | 2.2 | 2 | 1.4% |
|  | barking | | |  | | 0 | 0.0% | 0 | 0.0% | 0 | 0.0% | 1 | 0.7% |
|  | growling | | |  | | 4 | 3.4% | 7 | 5.4% | 10 | 7.2% | 9 | 6.4% |
|  | snapping | | |  | | 2 | 1.7% | 4 | 3.1% | 1 | 0.7% | 2 | 1.4% |
|  | biting | | |  | | 0 | 0.0% | 1 | 0.8% | 0 | 0.0% | 0 | 0.0% |
| **Behaviour during care and examination** | | | |  | | *n*=100 | | *n*=120 | | *n*=136 | | *n*=124 | |
| How does your dog behave when you  **brush him?** | | | |  | |  | |  | |  | |  | |
| acceptance | dog tolerates the situation |  |  | **3** | | 67 | 67.0% | 82 | 68.3% | 101 | 74.3% | 88 | 71.0% |
| slight withdrawal | dog tries to withdraw (movement of head or body) | | | **2** | | 22 | 22.0% | 29 | 24.2% | 25 | 18.4% | 27 | 21.8% |
| freeing | dog frees him-/herself from the owner’s fixation | | | **1** | | 3 | 3.0% | 7 | 5.8% | 6 | 4.4% | 5 | 4.0% |
| moves away | dog moves away from owner, owner cannot perform brushing | | | **0** | | 4 | 4.0% | 0 | 0.0% | 2 | 1.5% | 1 | 0.8% |
| aggression | dog barks or growls or bares teeth or snaps | | | **0** | | 4 | 4.0% | 2 | 1.7% | 2 | 1.5% | 3 | 2.4% |
| **examine his paws and teeth?** | | | |  | | *n*=144 | | *n*=149 | | *n*=147 | | *n*=147 | |
| acceptance | dog tolerates the situation |  |  | **3** | | 105 | 72.9% | 111 | 74.5% | 115 | 78.2% | 117 | 79.6% |
| slight withdrawal | dog tries to withdraw (movement of head or body) | | | **2** | | 28 | 19.4% | 25 | 16.8% | 26 | 17.7% | 20 | 13.6% |
| freeing | dog frees him-/herself from the owner’s fixation | | | **1** | | 6 | 4.2% | 5 | 3.4% | 2 | 1.4% | 1 | 0.7% |
| moves away | dog moves away from the owner, owner cannot perform examination | | | **0** | | 2 | 1.4% | 2 | 1.3% | 2 | 1.4% | 4 | 2.7% |
| aggression | dog bares teeth or barks or growls or snaps or bites | | | **0** | | 3 | 2.1% | 6 | 4.0% | 2 | 1.4% | 5 | 3.4% |
| **Reaction when leashed and walked.** | | | |  | |  |  |  |  |  |  |  |  |
| How does the dog behave when you **place the collar or harness?** | | | |  | | *n*=155 | | *n*=150 | | *n*=150 | | *n*=148 | |
| acceptance | dog tolerates the situation | | | **3** | | 98 | 63.2% | 113 | 75.3% | 126 | 84.0% | 122 | 82.4% |
| slight withdrawal | dog tries to withdraw (movement of head or body) | | | **2** | | 32 | 20.7% | 20 | 13.3% | 12 | 8.0% | 17 | 11.5% |
| moves away | dog moves away from owner, owner cannot place collar | | | **0** | | 22 | 14.2% | 14 | 9.3% | 11 | 7.3% | 9 | 6.1% |
| aggression | dog bares teeth or barks or growls or snaps or bites | | | **0** | | 3 | 1.9% | 3 | 2.0% | 1 | 0.7% | 0 | 0.0% |
|  | baring teeth | | |  | | 1 | 0.6% | 3 | 2.0% | 1 | 0.7% | 0 | 0.0% |
|  | barking | | |  | | 0 | 0.0% | 0 | 0.0% | 0 | 0.0% | 0 | 0.0% |
|  | growling | | |  | | 1 | 0.6% | 1 | 0.7% | 0 | 0.0% | 0 | 0.0% |
|  | snapping | | |  | | 3 | 1.9% | 2 | 1.3% | 1 | 0.7% | 0 | 0.0% |
|  | biting | | |  | | 0 | 0.0% | 0 | 0.0% | 0 | 0.0% | 0 | 0.0% |
| **lead your dog on a leash?** | | | |  | | *n*=155 | | *n*=151 | | *n*=151 | | *n*=149 | |
| follows along | dog follows along without the dog or the owner pulling on the leash | | | **3** | | 56 | 36.1% | 80 | 53.0% | 77 | 51.0% | 88 | 59.1% |
| moves after pulling | dog follows along only when the owner pulls on the leash | | | **1** | | 23 | 14.8% | 10 | 6.6% | 11 | 7.3% | 11 | 7.4% |
| pulls on the leash | dog pulls on the leash | | | **2** | | 49 | 31.6% | 50 | 33.1% | 50 | 33.1% | 44 | 29.5% |
| does not walk on the leash | even after pulling on the leash, the owner cannot make the dog walk, dog stops, sits or lies down | | | **0** | | 27 | 17.4% | 11 | 7.3% | 13 | 8.6% | 6 | 4.0% |
| **Unknown dog** |  | | | | | *n*=138 | | *n*=146 | | *n*=147 | | *n*=146 | |
| How does your dog behave when meeting another dog outside? | | | |  | |  |  |  |  |  |  |  |  |
| friendly contact | dog is wagging his/her tail, has relaxed body posture, plays with the other dog | | | **3** | | 52 | 37.7% | 64 | 43.8% | 60 | 40.8% | 72 | 49.3% |
| cautious contact | dog hesitantly approaches the other dog with signals of fear | | | **2** | | 24 | 17.4% | 20 | 13.7% | 24 | 16.3% | 10 | 6.8% |
| fear and avoidance | dog does not approach the other dog, dog moves away when being approached by the other dog and shows signals of fear | | | **0** | | 16 | 11.6% | 16 | 11.0% | 7 | 4.8% | 12 | 8.2% |
| does something else | dog does not seek contact and shows no change of current behaviour | | | **3** | | 12 | 8.7% | 15 | 10.3% | 17 | 11.6% | 19 | 13.0% |
| offensive aggression | dog approaches the other dog and bares teeth or barks or growls or snaps or bites | | | **0** | | 18 | 13.0% | 18 | 12.3% | 20 | 13.6% | 13 | 8.9% |

|  | baring teeth |  | 5 | 3.6% | 3 | 2.1% | 3 | 2.0% | 2 | 1.4% |
| --- | --- | --- | --- | --- | --- | --- | --- | --- | --- | --- |
|  | barking |  | 10 | 7.2% | 14 | 9.6% | 18 | 12.2% | 11 | 7.5% |
|  | growling |  | 10 | 7.2% | 9 | 6.2% | 12 | 8.2% | 3 | 2.1% |
|  | snapping |  | 2 | 1.4% | 2 | 1.4% | 1 | 0.7% | 1 | 0.7% |
|  | biting |  | 0 | 0.0% | 0 | 0.0% | 0 | 0.0% | 0 | 0.0% |

| defensive aggression | | dog barks or growls or bares teeth or snaps when being approached by the other dog | | **0** | | 14 | 10.1% | 12 | 8.2% | 16 | 10.9% | 18 | 12.3% |
| --- | --- | --- | --- | --- | --- | --- | --- | --- | --- | --- | --- | --- | --- |
|  | | baring teeth | |  | | 4 | 2.9% | 1 | 0.7% | 1 | 0.7% | 3 | 2.1% |
|  | | barking | |  | | 15 | 10.9% | 8 | 5.5% | 15 | 10.2 | 16 | 11.0% |
|  | | growling | |  | | 9 | 6.5% | 7 | 4.8% | 5 | 3.4% | 9 | 6.1% |
|  | | snapping | |  | | 2 | 1.4% | 1 | 0.7% | 1 | 0.7% | 3 | 2.1% |
|  | | biting | |  | | 0 | 0.0% | 0 | 0.0% | 0 | 0.0% | 1 | 0.7% |
| resource defence | | dog defends its food, toy or resting place with threatening or attacking behaviour or attacking behaviour or disputes with the other dog (bares teeth or barks or growls or snaps or bites) | | **0** | | 1 | 0.7% | 1 | 0.7% | 1 | 0.7% | 2 | 1.4.% |
|  | | baring teeth | |  | | 1 | 0.7% | 0 | 0.0% | 1 | 0.7% | 2 | 1.4% |
|  | | barking | |  | | 1 | 0.7% | 1 | 0.7% | 1 | 0.7% | 2 | 1.4% |
|  | | growling | |  | | 1 | 0.7% | 1 | 0.7% | 1 | 0.7% | 2 | 1.4% |
|  | | snapping | |  | | 0 | 0.0% | 0 | 0.0% | 0 | 0.0% | 1 | 0.7% |
|  | | biting | |  | | 0 | 0.0% | 0 | 0.0% | 0 | 0.0% | 0 | 0.0% |
| chasing | | dog stares at the other dog, runs after it and possibly snaps at it. | | **0** | | 1 | 0.7% | 0 | 0.0% | 2 | 1.3% | 0 | 0.0% |
| **Luring** | |  | |  | | *n*=155 | | *n*=151 | | *n*=151 | | *n*=149 | |
| How does the dog react to your attempts to make contact or lure it into the home, e.g. calling, snapping, whistling? | | | |  | |  | |  | |  | |  | |
| comes immediately | | dog comes directly to the owner without hesitation | | **3** | | 78 | 50.3% | 100 | 66.2% | 100 | 66.2% | 101 | 67.8% |
| comes hesitantly | | dog approaches the owner slowly and cautiously | | **2** | | 59 | 38.1% | 39 | 25.8% | 37 | 24.5% | 42 | 28.2% |
| does not come | | dog does not approach the owner | | **0** | | 18 | 11.6% | 12 | 7.9% | 14 | 9.3% | 6 | 4.0% |
| **Owner petting the dog**  How does your dog behave when you want to stroke or cuddle him? | | |  | | | *n*=155 | | *n*=151 | | *n*=151 | | *n*=149 | |
| enjoys | | dog wags tail/ rolls on back/ closes eyes/ rubs him-/herself against owner and so forth | | **3** | | 143 | 92.3% | 143 | 94.7% | 140 | 92.7% | 143 | 96.0% |
| acceptance | | dog tolerates the situation with tensed body | | **1** | | 5 | 3.2% | 3 | 2.0 % | 4 | 2.6% | 3 | 2.0% |
| slight withdrawal | | dog tries to withdraw (movement of head or body) | | **2** | | 1 | 0.7% | 3 | 2.0 % | 2 | 1.3% | 3 | 2.0% |
| moves away | | dog moves away from owner, owner cannot pet the dog | | **0** | | 5 | 3.2% | 2 | 1.3 % | 2 | 1.3 % | 0 | 0.0% |
| aggression | | dog bares teeth or barks or growls or snaps or bites | | **0** | | 1 | 0.7% | 0 | 0.0 % | 3 | 2.0 % | 0 | 0.0% |
|  | | baring teeth |  | | | 0 | 0.0% | 0 | 0.0% | 1 | 0.7% | 0 | 0.0% |
|  | | barking |  | | | 0 | 0.0% | 0 | 0.0% | 0 | 0.0% | 0 | 0.0% |
|  | | growling |  | | | 1 | 0.6% | 0 | 0.0% | 2 | 1.3% | 0 | 0.0% |
|  | | snapping |  | | | 0 | 0.0% | 0 | 0.0% | 2 | 1.3% | 0 | 0.0% |
|  | | biting |  | | | 0 | 0.0% | 0 | 0.0% | 0 | 0.0% | 0 | 0.0% |
| **Walk** | |  |  | | | *n*=155 | | *n*=151 | | *n*=151 | | *n*=149 | |
| Please describe the dog's behaviour outside (outside the home environment): | | |  | | |  | |  | |  | |  | |
| relaxed | | dog was/is mostly calm | | **3** | | 68 | 43.9% | 90 | 59.6% | 102 | 67.5% | 114 | 76.5% |
| excited | | dog was/is predominantly excited, constantly running around and panting a lot | | **2** | | 31 | 20.0% | 35 | 23.2% | 24 | 15.9% | 14 | 9.4% |
| anxious | | dog frequently shows signs of fear (e.g. trembling, crouched posture, tucked tail, attempts to escape) |  | | | 56 | 36.1% | 26 | 17.2% | 25 | 16.6% | 21 | 14.7% |
|  | Frequency of anxiety | | | | | | | | | | | | |
|  | | dog showed these signs of anxiety up to 5 times a day | | **1** | | 32 | 57.1% | 21 | 80.8% | 20 | 80.0% | 20 | 95,2% |
|  | | dog showed these signs of anxiety more than 5 times a day | | **0** | | 24 | 42.9% | 5 | 19.2% | 5 | 20.0% | 1 | 4,7% |
| **Playing** | | | |  | | *n*=155 | | *n*=151 | | *n*=151 | | *n*=149 | |
| plays | | dog follows the toy and/or picks up the toy with his/her mouth | | **-** |  | 76 | 49.0% | 92 | 60.9% | 99 | 65.6% | 107 | 71.8% |
| does not play | | dog does not engage in the play and does something else | | **-** |  | 79 | 51.0% | 59 | 39.1% | 52 | 34.4% | 42 | 28.2% |
| **Feeding out of hand (indoors)** | | | |  | | *n*=155 | | *n*=151 | | *n*=151 | | *n*=149 | |
| eats out of hand | | dog eats the food being offered out of the hand | | **-** |  | 146 | 94.2% | 149 | 98.7% | 148 | 98.0% | 148 | 99.3% |
| does not eat out of hand | | dog does not eat the food offered out of the hand | | **-** |  | 9 | 5.8% | 2 | 1.3% | 3 | 2.0% | 1 | 0.7% |
| **Feeding out of hand (outdoors)** | | | |  | | *n*=152 | | *n*=148 | | *n*=148 | | *n*=146 | |
| eats out of hand | | dog eats the food being offered out of the hand | | **-** |  | 125 | 82.2% | 133 | 89.9 % | 133 | 89.9 % | 139 | 93.3% |
| does not eat out of hand | | dog does not eat the food offered out of the hand | | **-** |  | 27 | 17.8% | 15 | 10.1 % | 15 | 10.1 % | 7 | 4.7 % |

**Table S2. To determine a possible influence of the placement procedure on the personality score of the dogs a placement process category was formed. The table shows the composition of this category from information from the first telephone interview before adoption or within the first week after adoption and the definitions of the respective criteria. The criterion ‘flow of information during placement process‘ was composed of four separate criteria. The mean value of all four criteria was formed.**

| **Definition criteria** | **Description** | | **Score** |
| --- | --- | --- | --- |
| **Meeting the dog in advance** |  | |  |
| The dog was met by the new owner before placement. | No | | 0 |
|  | Yes | | 1 |
| **Duration of consultation** |  |  |  |
| The duration of the consultation by the placement organisation was at least three hours. | No | | 0 |
|  | Yes | | 1 |
| **Flow of information during the placement process (did the organisation provide good information to the owner?)** | | |  |
| Information about the dog’s previous habituation abroad | No | | 0 |
|  | Yes | | 1 |
|  | **And** | |  |
| Information about the dog’s history before adoption | No | | 0 |
|  | Yes | | 1 |
|  | **And** | |  |
| Information about the dog’s pre-existing health conditions | No | | 0 |
|  | Yes | | 1 |
|  | **And** | |  |
| Information about the dog’s behaviour before adoption | No | | 0 |
|  | Yes | | 1 |

**Table S3.: Comparison of the mean values of the behaviour scales and the ‘manipulations’ score (components of personality score) of the telephone interviews from week one compared to month six. A One sample t-test of the differences was used for calculation with the alternative hypothesis: ‘true mean difference is not equal to 0’. Degrees of freedom (df).**

| **Value** | **t-value** | **Df** | **p-value** | **p-value adjusted** | **Mean of x** |
| --- | --- | --- | --- | --- | --- |
| Isolation | 3.898 | 108 | <0.001* | <0.01* | 0.61 |
| Contact with visitor | 0.743 | 117 | 0.459 | 0.653 | 0.11 |
| Passerby | 2.467 | 137 | 0.015* | 0.079 | 0.33 |
| Object first reaction | 3.03 | 148 | 0.003* | 0.019* | 0.38 |
| Object second reaction | 0.925 | 148 | 0.356 | 0.516 | 0.12 |
| Noise first reaction | 2.296 | 147 | 0.023* | 0.106 | 0.29 |
| Noise second reaction | 1.068 | 147 | 0.287 | 0.473 | 0.14 |
| Manipulations | 4.933 | 145 | <0.001* | <0.01* | 0.58 |
| Care by the owner | 0.387 | 90 | 0.699 | 0.832 | 0.04 |
| Examination by the owner | 1.505 | 136 | 0.135 | 0.284 | 0.12 |
| Placing collar or harness | 3.739 | 147 | <0.001* | <0.01* | 0.38 |
| Leash-Behaviour | 6.098 | 148 | <0.001* | <0.01* | 0.58 |
| Unknown Dog | 2.113 | 131 | 0.037* | 0.148 | 0.33 |
| Luring | 4.384 | 148 | <0.001* | <0.01* | 0.32 |
| Petting by owner | 2.505 | 148 | 0.013* | 0,078 | 0.13 |
| Walk | 8.011 | 148 | <0.001* | <0.01* | 0.70 |

*significant with p<0.05 **Table S4.: Improvement of the mean value of the personality score of the telephone interviews between 1 w. and 6 mos. A One sample t-test of the differences was used for calculation with the alternative hypothesis: ‘true mean difference is not equal to 0’. Degrees of freedom (df).**

| **Telephone interview** | **F-value** | **df** | **p-value** | **p-value adjusted** | **Mean of x** |
| --- | --- | --- | --- | --- | --- |
| 1w. to 6w. | 5.7294 | 150 | <0.001* | <0.01* | 0.199 |
| 6w. to 12w. | 1.7885 | 150 | 0.0757 | 0.212 | 0.050 |
| 12w. to 6mos. | 1.526 | 148 | 0.1292 | 0.278 | 0.043 |
| 1w. to 6 mos. | 8.4638 | 148 | <0.001* | <0.01* | 0.299 |

*significant with p<0.05

**Table S5. Willingness to adopt another dog in the future at telephone interview time 1w., 12w., 6mos.**

| **Parameter and Categories** | **Description or question asked** | **1w.  *n*=155** | | **12w. *n*=151** | | **6mos.**  ***n*=149** | |
| --- | --- | --- | --- | --- | --- | --- | --- |
| Would you choose a rescued dog from abroad again for adoption? | | No. | % | No. | % | No. | % |
| Yes | Adopt again | 131 | 84.5% | 124 | 82.1% | 119 | 79.9% |
| No |  | 5 | 3.2% | 10 | 6.6% | 10 | 6.7% |
| Only under certain conditions | By owners specified conditions see Table S6 | 19 | 12.3% | 17 | 11.3% | 20 | 13.4% |

**Table S6. Reasons why dog owners would decide against another dog and conditions under which dog owners would adopt a rescued dog from abroad again at telephone interview time 6mos.**

| **Reasons why dog owners would decide against another dog** | No.  *n*=10 | % |
| --- | --- | --- |
| dog is a surprise package | 6 | 60.0% |
| lack of socialisation of the dogs | 4 | 40.0% |
| transport was animal cruelty | 1 | 10.0% |
| own dog just a stroke of luck/lottery game | 3 | 30.0% |
| unknown health condition or hidden illnesses by animal welfare organisation | 3 | 30.0% |
| takes a lot of time/is exhausting to take care of unexperienced dog | 1 | 10.0% |
| it takes more time to get a dog used to living with humans than buying a puppy from a breeder. | 1 | 10.0% |
| dog does not come along with children/ grandchildren// less visits by grandchildren | 1 | 10.0% |
| financial aspects/high costs for illnesses of dog/owner has no money left for himself | 2 | 20.0% |
| overburdening of the owner | 1 | 10.0% |
| **Conditions under which dog owners would adopt again** | *n*=20 |  |
| ask more questions and gather more information beforehand especially about health condition and behaviour of the dog from the animal welfare organisation | 8 | 40.0% |
| get to know the dog beforehand in foster home or shelter in Germany | 6 | 30.0% |
| more transparency from the animal welfare organisation/only with better medical care, true information about illnesses and character of the dog and coverage of costs for imported diseases | 4 | 20.0% |
| only if it`s a puppy/young dog | 3 | 15.0% |
| only if dog was at a foster home before, so the dog could learn something | 1 | 5.0% |
| only if animal welfare organisation is reliable | 2 | 10.0% |
| not from this specific organisation (incorrect information about health) | 2 | 10.0% |
| only if it` no puppy/young dog | 1 | 5.0% |
| only if you have dog experience | 1 | 5.0% |
| only if dog had better socialisation | 1 | 5.0% |
| only if dog was healthy | 1 | 5.0% |
| breed-dependent, no longer a herding dog | 2 | 10.0% |
| only if small dog | 1 | 5.0% |
| only with no children in household | 1 | 5.0% |
| only if it is no stray dog | 1 | 5.0% |


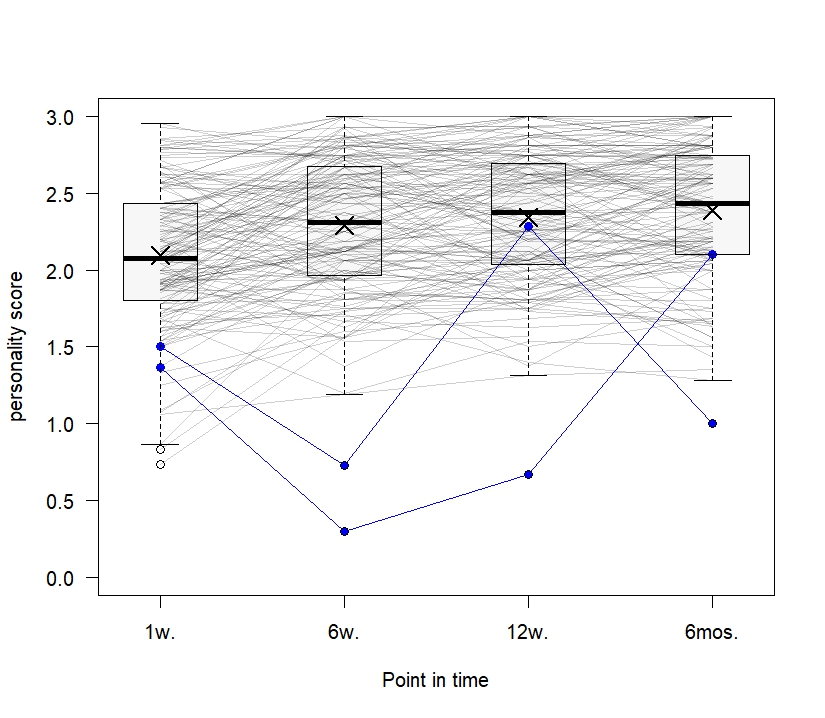


**Fig.S1. Personality scores from dogs (*n=149)* with data from all four telephone interviews (1w., 6w., 12w., 6mos.) after adoption. Every** l**ine marks the individual personality score development. Median marked as line and mean value as x. Two dogs were afraid to leave the house during all six months (see blue lines).**
